# How is Nanoarchitectonics Shaping Extracellular Vesicle Research?

**DOI:** 10.1002/adhm.71611

**Published:** 2026-08-31

**Authors:** Madushani Dahanayake, Md Shahriar A. Hossain, Chia‐Hung Liu, Carlos Salomon, Yusuke Yamauchi, Mostafa Kamal Masud

**Affiliations:** ^1^ Australian Institute of Bioengineering and Nanotechnology The University of Queensland Brisbane Queensland Australia; ^2^ School of Mechanical and Mining Engineering Faculty of Engineering Architecture and Information Technology (EAIT) The University of Queensland Brisbane Queensland Australia; ^3^ TMU Research Center of Urology and Kidney and Department of Urology School of Medicine College of Medicine Taipei Medical University Taipei Taiwan; ^4^ Department of Urology Shuang Ho Hospital Taipei Medical University New Taipei City Taiwan; ^5^ Translational Extracellular Vesicles in Obstetrics and Gynae‐Oncology Group Frazer Institute Faculty of Health, Medicine and Behavioural Sciences The University of Queensland Herston Queensland Australia; ^6^ UQ Centre for Extracellular Vesicle Nanomedicine The University of Queensland Brisbane Queensland Australia; ^7^ Department of Materials Process Engineering Graduate School of Engineering Nagoya University Nagoya Aichi Japan

**Keywords:** EV‐based diagnostics, EV engineering, extracellular vesicles, nanoarchitectonics, precision nanomedicine

## Abstract

Extracellular vesicles (EVs) have gained recognition as crucial mediators of cell‐to‐cell communication, holding immense potential in diverse biomedical applications. Nanoarchitectonics, a multidisciplinary approach encompassing nanotechnology and biomedicine, offers novel strategies for manipulating EVs at the nanoscale. This review explores the transformative influence of nanoarchitectonics on EVs research, focusing on the current progress in integrating various nanostructures to engineer EVs for enhanced therapeutic and diagnostic capabilities. The application of nanoarchitectonics has led to the fabrication of specialized nanostructures, such as plasmonic, fluorescent, magnetic, carbon‐based, and organic‐framework‐based nanostructures designed explicitly for EVs research. This approach enables the engineering of EVs with improved targeting specificity, cargo loading efficiency, and therapeutic efficacy, thus advancing their potential in precision medicine. The review addresses the opportunities and challenges associated with the convergence of nanoarchitectonics and EVs research, underscoring the necessity for further optimization, rigorous safety assessments, and interdisciplinary collaboration. Finally, the review concludes by discussing the strengths and limitations of the nanoarchitectonics–EVs interface in nanomedicine and therapeutics, providing a strategic roadmap for future advancements in this rapidly evolving field.

## Graphical abstract

The convergence of nanoarchitectonics and extracellular vesicle biology is reshaping next‐generation nanomedicine by enabling rationally designed nanostructures for enhanced EVs detection, engineering, and therapy.

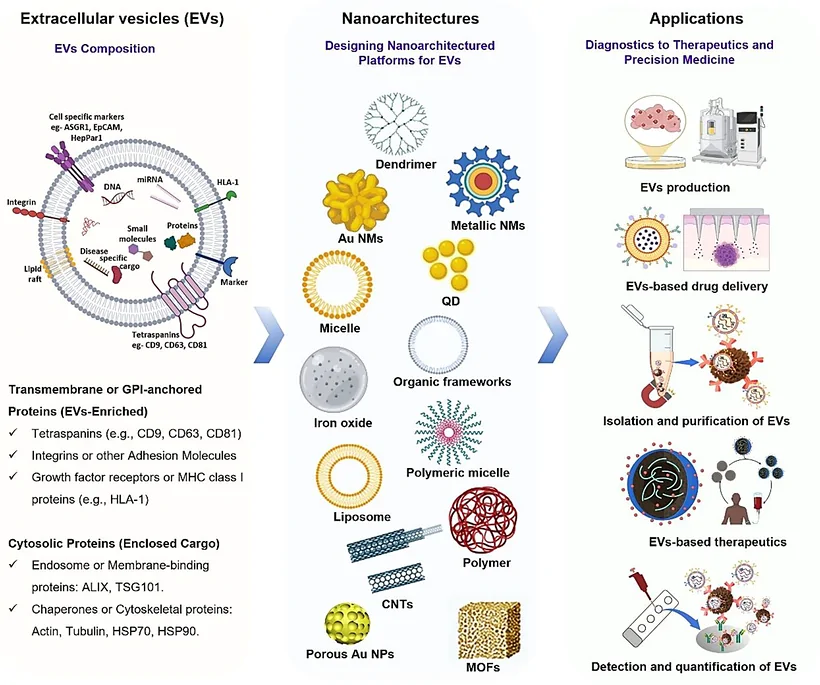

## 1 Introduction

Extracellular vesicles (EVs) have emerged as fundamental mediators of intercellular communication due to their ability to transport bioactive molecules such as proteins, lipids, and nucleic acids, including miRNAs, between cells [1, 2]. These nanoscale, lipid bilayer‐enclosed structures (approximately 40–1000 nm) participate in a wide range of physiological and pathological processes, positioning them as key targets in diagnostics, therapeutics, and drug delivery research [3]. EVs represent a heterogeneous population of vesicles, broadly classified into exosomes, microvesicles, microparticles, apoptotic bodies, and oncosomes, which differ in their biogenesis, size, and molecular composition [3, 4]. Exosomes arise from the endosomal pathway via multivesicular bodies (MVBs), which release intraluminal vesicles upon fusion with the plasma membrane [5, 6]. MVBs encapsulate cytoplasmic material and cell‐surface proteins, helping to mediate signal transduction and modulation of the extracellular environment. These vesicles are key players in inflammatory responses and pathological processes, including cancer and cardiovascular diseases. Fusion of MVBs with the plasma membrane releases intraluminal vesicles into the extracellular space, thereby generating exosomes [7]. In contrast, microvesicles and related subtypes originate from direct outward budding of the plasma membrane, often reflecting cellular activation or pathological stress [8]. Microparticles are released by a variety of cells in response to stress, activation, or injury and are involved in cell signaling, inflammation, and coagulation. They carry surface markers and bioactive molecules from their parent cells, influencing processes such as immune response and thrombosis [9]. Apoptotic bodies serve primarily to package and dispose of cellular debris, whereas their clearance by phagocytes prevents inflammation and contributes to immune tolerance and tissue homeostasis [10]. Oncosomes are shed specifically by cancer cells, especially those undergoing oncogenic transformation, as they carry oncogenic proteins, mutated DNA, and signaling molecules that promote tumor growth, invasion, and metastasis. Oncosomes play a role in modifying the tumor microenvironment and have the capacity to serve as biomarkers for cancer progression [11]. Among all EV subtypes, exosomes have garnered considerable attention owing to their unique biogenesis, selectively incorporating cytosolic and membrane components. These nanoscale vesicles are capable of interacting with adjacent cells or relocating to distant targets, facilitating communication and modulating various physiological processes. Upon reaching their destination, exosomes deliver a cargo of miRNAs, mRNAs, proteins, and lipids, inducing significant changes in the behavior and function of recipient cells [3, 12]. The growing interest in EVs, particularly their miRNA profiles, has highlighted their potential as both biomarkers and therapeutic targets [13]. Researchers are actively exploring methods to engineer EVs for enhanced loading of cargo and targeted delivery of therapeutic agents and imaging tracers [14]. Recent investigations have shown that EVs play a critical role in maintaining neural plasticity, neurovascular integrity, and brain homeostasis, while also contributing to the progression of neurological disorders such as stroke and neurodegenerative diseases [15]. These findings underscore the immense diagnostic and therapeutic potential [16].

Despite explosive growth in EV research, translation into clinically robust technologies remains severely constrained. Major bottlenecks persist in scalable production, purification fidelity, molecular heterogeneity, unstable circulation behavior, inefficient cargo loading, and poor control over targeting specificity. In parallel, the extreme nanoscale dimensions and low analyte abundance of EVs continue to challenge conventional analytical platforms such as ELISA, PCR, Western blotting, and flow cytometry, limiting their sensitivity, reproducibility, and clinical scalability [17, 18]. Although emerging nanotechnology‐based tools have partially improved detection capability, a fundamentally new design paradigm is required to move beyond incremental progress. Nanoarchitectonics has emerged as such a paradigm‐shifting framework, fundamentally redefining how EV systems are conceptualized, engineered, and applied. By integrating nanotechnology with chemistry, materials science, and biological self‐organization principles, nanoarchitectonics enables the construction of functional systems through controlled hierarchical assembly, molecular precision, and emergent nanoscale interactions [19, 20]. Crucially, this approach shifts EVs research from passive observation to active structural engineering, where biological function is no longer merely interpreted but rationally designed through nanoscale architecture. Within this framework, EVs are no longer treated as static biological carriers but as programmable nanostructures whose behavior is governed by membrane organization, molecular composition, and mechanical properties. EV membrane stiffness, elasticity, and curvature have emerged as decisive nanoarchitectural parameters that dictate circulation stability, barrier penetration, cellular uptake, intracellular trafficking, and cargo release [21]. These mechanical and structural determinants open a new dimension of EV engineering, enabling functional reprogramming through lipid remodeling, membrane reorganization, and nanomaterial integration. This represents a conceptual shift from biochemical modulation to mechanics‐driven control of biological function.

More importantly, nanoarchitectonics enables the creation of next‐generation hybrid EVs‐nanomaterial systems that merge biological intelligence with synthetic functionality [22, 23]. In these architectures, EVs provide intrinsic biocompatibility and targeting capability, while engineered nanomaterials introduce optical, magnetic, and catalytic functionalities, collectively driving a paradigm shift in drug delivery and therapeutics through nanoarchitectonics‐enabled systems with unprecedented potential for precision medicine and personalized treatments [24, 25]. For example, hybrid EV‐nanomaterial systems can be engineered to combine the intrinsic targeting and biocompatibility of EVs with the enhanced stability and functional properties of nanoparticles (NPs). This synergy improves therapeutic efficacy while reducing off‐target effects. In cancer therapy, EVs loaded with chemotherapeutic agents and functionalized with gold nanoparticles (AuNPs) enable combined chemo‐photothermal treatment, where near‐infrared (NIR) irradiation triggers localized hyperthermia to potentiate drug cytotoxicity within the tumor microenvironment [26]. In neurological applications such as Alzheimer's disease, nanoarchitectonics‐enabled EV systems integrated with magnetic nanoparticles (MNPs) show enhanced blood–brain barrier (BBB) penetration and enable targeted delivery to the brain. The incorporation of MNPs further allows non‐invasive magnetic resonance imaging (MRI) tracking, enabling real‐time monitoring of biodistribution and therapeutic progression [27]. Beyond delivery, meso‐ and nanoporous nanostructures, particularly magnetic materials, are increasingly used for EV isolation and analysis due to their high capture efficiency and facile magnetic separation [6, 28].

**FIGURE 1:**
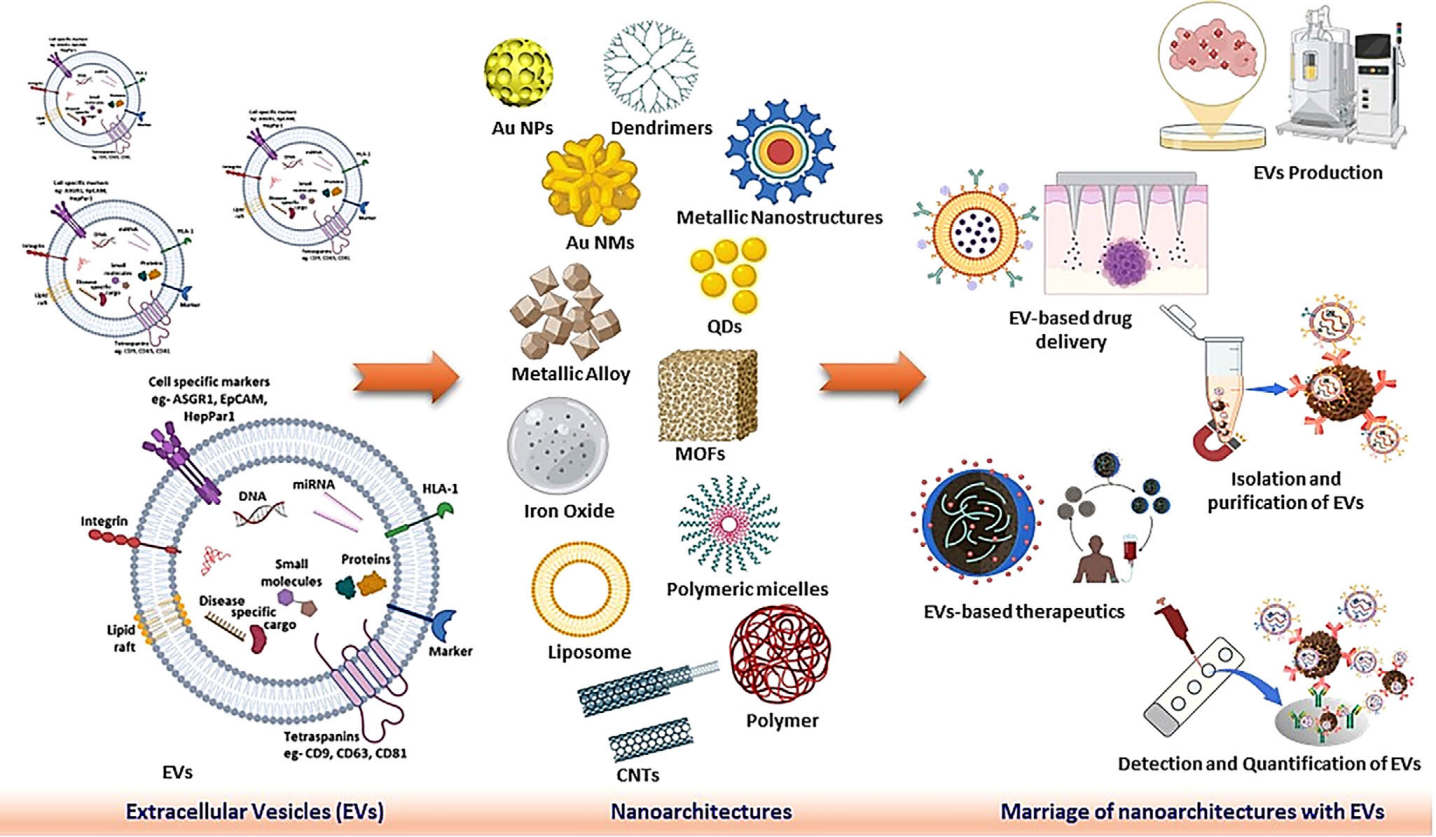
Nanoarchitectonics is reshaping extracellular vesicle research from biological discovery to precision nanomedicine. Exosome biogenesis governs EV formation, cargo composition, and functional diversity. Rationally engineered nanoarchitectures provide advanced tools for EV capture, analysis, modulation, and translation. By integrating nanoscale design with EV biology, nanoarchitectonics enables next‐generation platforms for diagnostics, therapeutics, and personalized medicine.

Despite these advances, a unified understanding of how nanoarchitectonics systematically governs EV production, engineering, and function remains incomplete. Addressing this gap is essential for translating EV‐based technologies into reliable clinical platforms. Therefore, this review discusses how nanoarchitectonics is shaping EV research across multiple dimensions, including EV biogenesis, isolation, detection, engineering, and therapeutic applications (Figure 1). By linking nanoscale design principles with EV function, we highlight how nanoarchitectonics transforms EV research from a biologically descriptive field into a rational, design‐driven discipline, with significant implications for precision medicine.

## 2 Extracellular Vesicles

The role of EVs was initially uncertain, whether they served as a mechanism for regular cells to remove unnecessary material and maintain tissue balance or as a tool used by cancer cells to drive tumor growth and metastasis [29]. However, current understanding has evolved, recognizing the diverse and crucial roles that EVs play in intercellular communication. These vesicles facilitate communication by either transferring their internal cargo to recipient cells upon uptake or through interactions between surface proteins on the EVs and cellular receptors. This communication is essential for maintaining normal physiological functions [30]. EVs are particularly notable for their ability to carry bioactive molecules, mainly proteins, lipids, DNA, and RNA, especially miRNAs, positioning them as valuable biomarkers and therapeutic targets [31, 32]. Their biocompatibility, ability to cross biological barriers, and intrinsic targeting capacities make them ideal candidates as disease biomarkers and drug delivery vehicles. EVs occur in several types categorized by their origin and methods of formation. Three major modes of biogenesis are identified, namely apoptotic EVs, ectosomes, and exosomes. As EV composition depends strongly on their cell of origin and physiological state, their functions vary widely and can be broadly divided into disease‐associated and therapeutic roles (Table 1). In addition, given the variety of EV types and uncertainties regarding their biogenesis, the Minimal Information for Studies of Extracellular Vesicles (MISEV) has categorized EVs practically as either ‘large EVs’ (size > 200 nm) or ‘small EVs’ (size < 200 nm) [33]. Among these, exosomes are the most extensively studied, primarily because of their unique biogenesis. They are formed within multivesicular endosomes (MVEs) through an inward‐budding process at the late endosomal membrane, creating intraluminal vesicles (ILVs) [34, 35]. These ILVs are ultimately released as exosomes (30–150 nm) upon fusion of MVEs with the plasma membrane. The process incorporates the Endosomal Sorting Complex Required for Transport (ESCRT) machinery, which sorts proteins into ILVs. While traditionally associated with exosome biogenesis, several proteins involved in this process, such as TSG101 and ALIX, also play roles in outward budding from the plasma membrane [31, 32]. Additionally, small EVs containing tetraspanins (TSPANs) like CD63, CD81, and CD9 were initially considered exosome markers, but they can originate directly from the plasma membrane, categorized as ectosomes or microvesicles [36, 37]. The RAB class of small G proteins, notably RAB7, RAB11, as well as RAB35, regulates secretion and trafficking of exosomes, whereas RAB27A and RAB27B facilitate the release of ILVs as exosomes. Membrane contact sites between of endoplasmic reticulum (ER) and endosomes, facilitated by RAB7, are implicated in exosome biogenesis [38]. Additionally, some RAB proteins like RAB13 and RAB31 are involved in the release of specific groups of EVs or in alternative pathways of exosome biogenesis [39].

**TABLE 1:** Classification of EVs and their functional roles.

EV type	Biogenesis	Size range	Typical cargo	Physiological roles	Nanomedicine relevance	Ref.
Small EV	Released via fusion of multivesicular bodies with the plasma membrane.	30–150 nm	Tetraspanins (CD9, CD63, CD81), Alix, TSG101, mRNA, miRNA, lipids.	Intercellular communication, regulation of gene expression and signaling.	Biomarkers Drug delivery vehicles Regenerative therapy tools	[45, 46]
Ectosomes (microvesicles)	Direct outward budding from the plasma membrane.	100–1000 nm	Membrane proteins, cytosolic proteins, mRNA, miRNA, lipids.	Rapid transfer of surface receptors and signaling molecules between cells.	Disease biomarkers Modulators of inflammation and thrombosis	[47, 48]
Apoptotic bodies	Released during programmed cell death (apoptosis).	1–5 µm	DNA fragments, organelles, histones, cellular components.	Clearance of dying cells may also mediate immune and inflammatory signaling.	Potential biomarkers in cancer, autoimmune, and inflammatory diseases	[49, 50]

**FIGURE 2:**
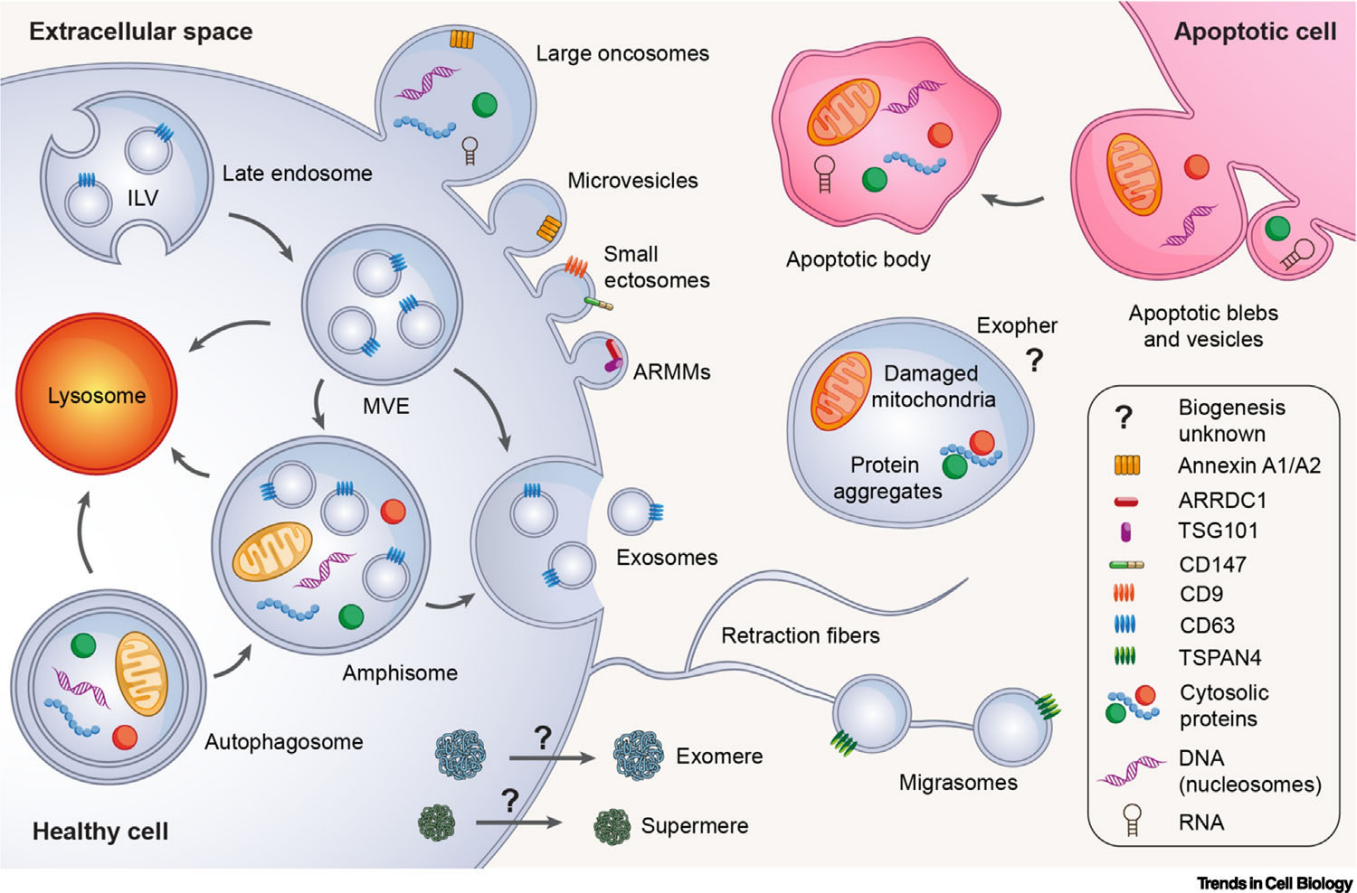
Ectosomes (30 nm–10 µm) form through outward budding of the plasma membrane and include microvesicles, large oncosomes (malignant cells), small ectosomes, and ARMMs. Apoptotic bodies/vesicles (range from 100 nm to 5 µm) are released during apoptosis. Intraluminal vesicles (ILVs) form via inward budding of late endosome membranes, creating multivesicular endosomes (MVEs), which release ILVs as exosomes (30–120 nm) or fuse with autophagosomes. Migrasomes (500 nm–3 µm) are released from migrating cell retraction fibers. Exophers (3.5–4 µm) consist of damaged mitochondria and protein aggregates, released under stress. Exomeres and supermeres (20–50 nm) are non‐membranous extracellular nanoparticles, biogenesis unknown, found in circulation from normal and cancer cells. Reproduced with permission from Ref. [29]. Copyright 2023, Elsevier.

Ectosomes, generated via outward budding process of plasma membrane, were initially thought to be large EVs but can also exist as small EVs (<200 nm) [30]. They encompass various subtypes, including classical microvesicles and small ectosomes. Microvesicles are characterized by Annexin A1 and A2 expression and can range from 150 nm to 1000 nm, while small ectosomes like ARMMs and synaptic ectosomes have distinct protein markers and functions [40]. The biogenesis of microvesicles involves rearrangement of the actin cytoskeleton, regulated by ARF6, and can vary in size and cargo depending on the cellular context [41]. Apoptotic EVs, released during apoptosis, include apoptotic bodies and vesicles, distinguished by their size and molecular cargo. Apoptotic bodies are larger (1–5 µm) and contain histones, DNA, and cellular organelles, while apoptotic vesicles are smaller (100–1000 nm) and express PS on their outer leaflet [42, 43]. Other types of EVs, such as migrasomes, exophers, and autophagy‐related EVs, have been identified, each with specific biogenesis pathways and functions (Figure 2) [24]. Owing to their exceptional attributes, EVs are emerging as a class of analytes for liquid biopsy for diagnostics, prognosis, and treatment monitoring of various diseases, especially cancer. EVs are constantly discharged into the extracellular environment by almost all cell types, with exosomes originating from endosomes, whereas microvesicles from the plasma membrane. These tiny particles carry genetic material and a range of proteins, such as tetraspanins, receptors, and adhesion molecules, reflecting the molecular features of their parental cells [44]. EVs derived from tumor cells have the ability to promote carcinogenesis and progression via regulating angiogenesis, immunology, and metastasis, as well as transferring oncogenic substances through intercellular communication. Tumor‐derived EVs circulate in blood and other biofluids, providing a robust reservoir of biomarkers for noninvasive liquid biopsy in medicine [29]. A summary of the functional roles of therapeutic and disease‐associated EVs is provided in Table 2.

**TABLE 2:** Functional classification of EVs based on biological roles.

EVs	Origin	Key molecular cargo	Main functions	Applications	Ref.
Disease‐associated EVs	Tumor cells	VEGF, MMPs, oncogenic miRNAs, mutant DNA, immunosuppressive proteins	Promote angiogenesis, metastasis, extracellular matrix remodeling, immune evasion	Cancer diagnosis and monitoring (liquid biopsy) Therapeutic targets in cancer	[51, 52]
	Inflamed or stressed cells	Cytokines, lipids, DAMPs, pro‐coagulant factors	Amplify inflammation, endothelial dysfunction, thrombosis	Biomarkers in cardiovascular, pulmonary, and neurodegenerative diseases	[53]
	Pathogen‐infected cells	Viral RNA/DNA, microbial proteins, toxins	Modulate host immunity, enhance pathogen spread or immune escape.	Infectious disease diagnostics	[54]
Therapeutic EVs	Mesenchymal stem cells (MSCs)	Regenerative miRNAs, growth factors, anti‐inflammatory proteins	Promote tissue repair, angiogenesis, immune modulation.	Wound healing, cardiac and bone regeneration	[55]
	Engineered cells	siRNA, miRNA, drugs, CRISPR components	Targeted delivery of therapeutic cargo.	Cancer therapy Gene therapy	[56]
	Dendritic cells	MHC–peptide complexes, co‐stimulatory molecules	Activation of antigen‐specific immune responses.	EV‐based vaccines and immunotherapies	[57]
	Engineered receptor‐expressing EVs	Surface receptors (e.g., ACE2)	Act as decoys to neutralize pathogens.	Antiviral decoy therapies	[58]

## 3 Roles of Nanoarchitectonics on EVs Research

EVs have emerged as biologically significant carriers of molecular information, with their diverse biogenesis pathways and cargo profiles making them promising targets for cancer diagnostics and therapeutics. However, their small size, structural fragility, heterogeneity, and low abundance in biological fluids present major challenges for efficient isolation, characterization, and clinical application. Nanoarchitectonics provides a powerful design framework to address these limitations by integrating nanoscale building blocks into functional architectures that can interact with EVs in a precise and controllable manner. The fundamental design principles of nanoarchitectonics in EVs research involve tailoring nanomaterials according to the unique physicochemical characteristics of EVs, including their nanoscale dimensions, lipid bilayer structure, surface proteins, and molecular cargo. By controlling material composition, morphology, surface chemistry, and hierarchical organization, nanoarchitected systems can be engineered to selectively capture, enrich, detect, track, or modify EVs. High surface‐area nanomaterials with tunable surface functionalities enable enhanced EVs binding and biomarker recognition, while biomimetic interfaces facilitate membrane interactions, cargo loading, and targeted delivery. Such rational material design allows seamless integration of nanostructures into EVs‐based diagnostic and therapeutic platforms.

Recent developments have demonstrated the successful integration of nanomaterials with EVs to create hybrid systems that exhibit enhanced therapeutic and diagnostic performance compared with either component alone [19]. Nanostructures can be engineered to impart specific functionalities, including photothermal therapy, magnetic hyperthermia, molecular imaging, and targeted drug delivery, thereby expanding the theranostic capabilities of EVs‐based platforms [59, 60, 61]. Conversely, EVs provide a biologically compatible carrier that protects nanostructures from immune clearance and facilitates transport across biological barriers, including the blood–brain barrier. In addition, EVs offer a versatile bioengineering platform for gene delivery and functional modification. For example, electroporation can transiently permeabilize the EV membrane, enabling efficient loading of nanomaterials and therapeutic cargo. Advances in nanotechnology, particularly in microfluidics and nanoscale engineering, have further improved EVs synthesis, isolation, characterization, and detection, addressing key challenges related to scalability, sample volume, and analytical sensitivity [62, 63, 64, 65]. These advancements hold transformative potential for EVs‐based diagnostics and theranostic applications, supporting future precision‐medicine applications.

### 3.1 EVs Production

The initial step in employing EVs as therapeutic vehicles for diverse diseases involves isolating/collecting EVs from culture media or biofluids. However, several challenges hinder EV preparation from meeting clinical requirements [66]. Firstly, scalable EVs production is necessary to yield higher quantities for targeted delivery. Secondly, both unmodified and modified EVs must adhere to high‐quality standards with standardized protocols for practical applications. Over the past decade, various approaches have been developed by scientists to enhance EVs biogenesis and discharge from cultured cells by incorporating genetic‐engineering, stress‐induction, chemical regulation, or physical methods. Genetic‐engineering methods, which employ engineered genetic tools targeting multiple regulation pathways, have shown the potential to increase EVs yield significantly [67]. However, the complexity and expense of modulating donor cells, coupled with the risk of unexpected mutations, pose challenges. Stress‐inducing conditions (e.g., hypoxia, acidic pH levels, and starvation) and chemical regulators (e.g., mephenesin and norepinephrine) offer convenient methods for large‐scale stimulation of donor cells but typically induce only modest increases in EVs release (1.4–5.7‐fold) [68, 69]. Conversely, certain biological functions such as regeneration and anti‐inflammatory responses can be enhanced through exposure to stressors or specific compounds. Similarly, physical methods such as electric fields, ultrasound, mechanical forces, or ionizing radiation can moderately increase EVs release (<10‐fold) but require costly instrumentation and may compromise cell integrity [70, 71].

Nanostructures have recently been explored to accelerate EV manufacturing without compromising donor‐cell integrity. The structural, mechanical, and chemical characteristics of nanostructures can actively modulate signaling pathways in these cells and induce EV secretion [19]. For instance, nanostructures can influence EV production, as they have the ability to modulate the intracellular vesicular system. Upon entry into mesenchymal stem cells, a positively charged nanoparticle is transported to lysosomes, initiating the secretion of EVs by stimulating autophagy‐related dynamics [72]. A study by Shyong et al. confirmed that upon the release of Ca^2+^ into cytosol, calcium phosphate (CaP) particles could significantly increase exosome secretion by more than two‐fold. Furthermore, the surface topography of nanostructures can also influence EV biogenesis and generate bioactive signals [73]. For example, a micro‐/nano‐textured hierarchical topography has been shown to promote exosomal secretion by upregulating the expression of SMPD3 and RAB27B [74]. These findings underscore the potential of nanostructures in facilitating efficient EV production, particularly for drug delivery applications. The advantages of incorporating nanostructures include ensuring safety with insignificant changes in cell integrity, while also potentially enhancing the presence of desired bioactive molecules within EVs. Moreover, the combination of nanomaterials with bioreactor systems offers a convenient pathway for large‐scale EV manufacturing. Nevertheless, this strategy faces challenges such as limited enhancement of EV release. Consequently, there is a continued need to develop nanomaterials with efficient stimulating effects while elucidating the underlying mechanisms involved. Importantly, beyond production efficiency, the mechanical properties of EVs, particularly their stiffness are emerging as crucial indicators of their quality and functional potential [75]. Studies have shown that EV stiffness, which can be modulated by altering the biophysical environment of donor cells or incorporating nanomaterials, plays a role in influencing biodistribution, cellular uptake, and therapeutic efficacy. Softer EVs tend to exhibit enhanced fusion and internalization by target cells, while stiffer EVs may have improved circulation stability. Thus, optimizing EV stiffness during production is a promising strategy to tailor EVs for specific clinical applications.

**FIGURE 3:**
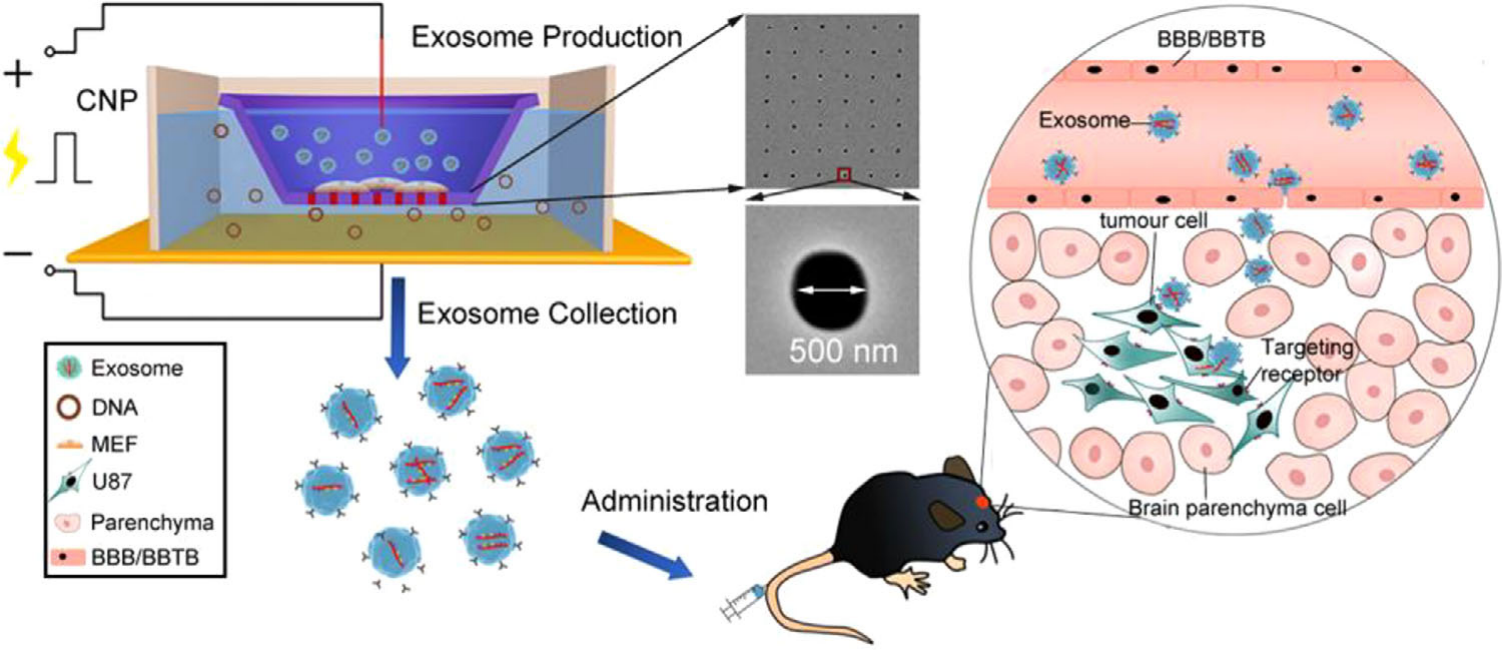
Schematic illustration of EVs produced via cellular nanoporation (CNP) for targeted delivery of nucleic acids. The CNP system comprises a nanochannel array (depicted as red rectangles), with each nanochannel measuring up to 500 nm in diameter (top inset). Plasmid DNA introduced into the buffer enters adherent cells through the nanochannels under transient electrical pulses. The transfected cells subsequently release abundant exosomes containing the transcribed mRNA, which can be harvested for targeted delivery across the blood–brain barrier (BBB) or blood–brain tumor barrier (BBTB) (right). Reproduced with permission from Ref. [80]. Copyright 2019, Springer Nature.

To address the limitations of large‐scale manufacturing while increasing the yield of EVs, methods involving the fabrication of EV‐like nanovesicles have been developed. These techniques include sonication, nitrogen cavitation, dissolution in high pH environments, extrusion through porous membranes, or biomimetic artificial synthesis [76]. These approaches involve disintegrating cells and reconstituting the resulting cellular components to produce nanovesicles. The yield of EV‐like nanovesicles is typically tens to hundreds of times more efficient in comparison to naturally secreted EVs, subjected to the specific technique employed. However, despite the advantages of EV‐like nanovesicles in terms of yield, they may lack the therapeutic content present in natural EVs, potentially compromising their therapeutic efficacy [77, 78, 79]. Additionally, EV‐like nanovesicles contain numerous cytoplasmic constituents, which could lead to unforeseen effects such as immune responses and tumorigenicity in vivo. Yang et al. introduced a cellular nanoporation (CNP) biochip featuring a nanochannel array for the mass production of exosomes [80]. These nanochannels, approximately 500 nm in diameter, provided focal and transient electric stimulation to attached cells, resulting in a significant increase in both exosome release (50‐fold increase) and the loading of transcribed mRNAs (1000‐fold increase). mRNA‐encapsulated exosomes were capable of effectively restoring tumor‐suppressor function in a glioma mouse model that was PTEN‐deficient (Figure 3). While this cellular nanoporation method offers a means to achieve large‐scale EVs production with minimal alteration to cell integrity, challenges remain, including the engineering of such biochips and maintaining consistent EVs production. Nevertheless, this approach offers convenience and can be integrated with microfluidics for scaled‐up generation. While promising, these strategies are still in early development and require further refinement before they can be applied clinically. The future of EVs‐based therapies lies in the ability to produce uniform, therapeutically potent EVs on a large and cost‐effective scale. Overall, nanoarchitectonics is expanding the toolbox for scalable and functional EVs production; however, current approaches remain constrained by limited standardization and translational readiness. Addressing downstream challenges in EVs isolation and purification is therefore equally important for realizing clinically relevant EVs technologies.

### 3.2 Drug Delivery

EVs including exosomes, microvesicles, and apoptotic bodies, have garnered attention as useful carriers for drug delivery, particularly in cancer therapy and other medical conditions [81]. These vesicles excel in intercellular communication, leveraging their membrane‐bound structures enriched with adhesive proteins to transport therapeutic cargo directly into target cells [82]. The bilayer membrane of EVs allow controlled and sustained drug release, improving stability and solubility while safeguarding cargo from degradation in the bloodstream. Importantly, EVs exhibit low immunogenicity and cytotoxicity, which makes them a safe, non‐toxic option for therapeutic use, especially when derived autologously [83]. An emerging aspect of EV functionality in drug delivery is the mechanical property of EV stiffness. EVs' stiffness plays a crucial role in determining their uptake by recipient cells, biodistribution, and ability to navigate physiological barriers. Stiffer EVs are often associated with reduced cellular uptake, while softer EVs display enhanced membrane fusion, improving internalization efficiency [84]. Therefore, engineering EVs' stiffness through lipid composition modulation or cargo loading strategies could optimize their therapeutic performance, tailoring them for specific clinical applications. Among all EVs, exosomes, commonly found in bodily fluids like blood, urine, and culture media, offer exciting potential when combined with nanostructures [44]. By fusing with these nanostructures, exosomes gain enhanced targeting precision, increased permeability, and reduced toxicity, all while benefiting from the enhanced permeability and retention (EPR) effect. Originating from specific cell types, exosomes effectively transfer molecular cargo, such as RNA, microRNA, and chemotherapeutics, between cells [85, 86]. They can be loaded with drugs and NPs to augment targeting abilities, effectively delivering RNA, small interfering RNA, microRNA, and chemotherapeutics for enhanced cancer treatment. Despite these advantages, challenges such as isolation, EV heterogeneity, and characterization impede their widespread use, particularly in clinical settings. Further studies are needed to improve their stability and loading capacity during drug‐loading and surface‐modification processes [44].

Nanostructures have long been utilized in clinical drug delivery, offering enhancements in stability, solubility, and circulation time [87]. These engineered NPs, equipped with targeting agents, have been pivotal in improving cell‐specific targeting and intracellular drug transport [88]. However, synthetic nanostructures face limitations, such as nonspecific molecular targeting and poor bioavailability, which can lead to inefficiencies in clinical trials [89]. To address these hurdles, a hybrid approach that combines EVs and nanostructures is proposed. EVs‐coated nanostructures bypass immune responses, offer superior tumor targeting, and exhibit better drug‐loading capabilities than purely synthetic vectors. While EVs variability based on donor cells remains a challenge, these hybrid systems show immense promise in clinical applications, particularly for personalized therapies. This multifunctional carrier offers diverse sizes and shapes relying on drug conjugation or encapsulation abilities. However, EVs composition variability based on donor cell sources influences drug targeting. Despite ongoing clinical trials, EVs, especially exosomes, show promise as drug/gene carriers, suggesting their potential therapeutic efficacy in personalized medicine. Furthermore, integrating stiffness tuning into EVs‐nanostructure hybrid platforms may amplify drug delivery efficacy [90]. By modulating mechanical stiffness, researchers can design EVs optimized for different tissue environments or pathological conditions. For example, softer EVs may be more effective for penetrating tumor tissues or the blood–brain barrier, while stiffer EVs might exhibit longer circulation times.

In recent years, nanostructures have emerged as promising carriers for drugs and therapeutic agents. Nanostructures offer controllable structure, composition, and pore size, allowing for tailored applications. To enhance interactions with biological structures, various types of functional groups can be introduced to their exterior and interior surfaces. NPs possess a large surface area and high porosity, facilitating high drug loading capacity and controlled release. By combining nanostructures with exosome shells, a novel drug delivery platform with unique features such as highly ordered porous networks, size homogeneity, and customizable surfaces is envisioned. These hybrid structures hold promise for personalized medicine and targeted therapies, offering enhanced efficacy, specificity, and safety profiles. However, challenges such as selecting specific EVs for coating, efficient encapsulation of exosomes, stability in bodily fluids, and engineering nanostructures for desired properties need to be addressed for clinical translation. Collaborative efforts among researchers, clinicians, and industry stakeholders are crucial for advancing these technologies and improving patient outcomes.

In recent years, MOFs have emerged as versatile carriers for drugs or therapeutic agents, offering controllable composition, structure, and pore size ideal for tailored drug delivery applications [91, 92, 93, 94]. MOFs can be modified with functional groups on their internal and external surfaces to enhance interactions with biological structures, allowing for high drug loading capacity. Illes et al. demonstrated the synergistic potential of MOFs and exosomes for targeted drug delivery by coating iron oxide‐based MOFs with exosomes obtained from HeLa cell culture, resulting in effective drug release and significant cell death [95]. Similarly, Cheng and colleagues synthesized exosome‐coated protein‐loaded MOF nanostructures, showing promise for tumor targeting and suppression [96]. Nanosized MOFs (NMOFs) have also shown potential for gene delivery, enhancing therapeutic efficacy in drug‐resistant cancer cells [97]. However, addressing challenges such as efficient encapsulation and stability in bodily fluids is crucial for clinical translation. Collaborative efforts are essential for advancing MOF‐based drug delivery systems and realizing their therapeutic potential in clinical practice. Recent studies have explored combining Au nanostructures, especially AuNRs, with exosomes for the purpose of tumor‐targeted chemo‐photothermal therapy [98, 99]. By attaching AuNRs to exosomes, localized laser‐induced hyperthermia enhances exosome membrane instability, facilitating drug encapsulation and selective tumor cell destruction. Further functionalization of the surface of these AuNR‐coated exosomes with targeting ligands enhances tumor cellular uptake and therapeutic outcomes. In another approach, nanovehicles comprising Au NPs, exosomes resembling passion fruit, and photosensitizer molecules were developed for real‐time fluorescence imaging, as well as targeted photodynamic therapy [100]. These nanovehicles efficiently targeted tumor cells, penetrated deeply, and released photosensitizer molecules upon laser irradiation, effectively inhibiting tumor growth.

**FIGURE 4:**
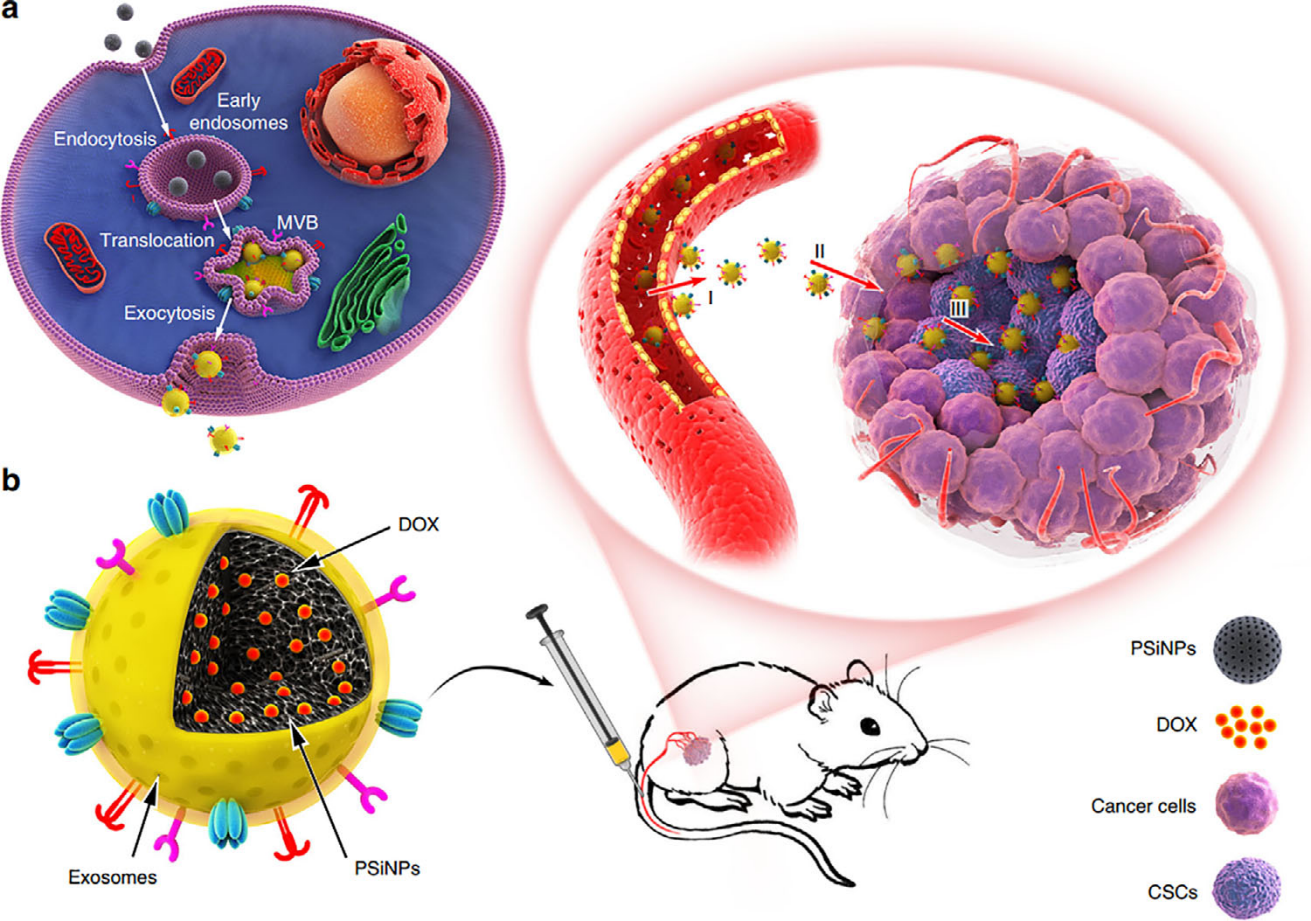
Illustration depicting exosome‐sheathed porous silicon nanoparticles (E‐PSiNPs) as carriers for targeted cancer chemotherapy. (a) Doxorubicin‐loaded E‐PSiNPs (DOX@E‐PSiNPs) are internalized into cancer cells via endocytosis after incubation, subsequently localizing in multivesicular bodies and autophagosomes. (b) Upon intravenous administration of DOX@E‐PSiNPs into tumor‐bearing mice, the substances effectively accumulate in cancer tissues, penetrating deeply into tumor parenchyma to be internalized into bulk cancer cells and cancer stem cells, leading to potent anticancer effects. Reproduced with permission from Ref. [1]. Copyright 2019, Springer Nature.

Superparamagnetic iron oxide nanostructures (SPIONs) offer precise drug delivery with external magnets [101, 102, 103, 104]. They show promise in cardiovascular diseases, with MSC‐derived exosomes enhancing cardiac repair. SPION‐based exosome platforms also target cancer cells, combining with TNF‐α for enhanced therapy and overcoming the blood–brain barrier in glioma treatment. These systems improve drug retention, reduce side effects, and enhance therapeutic efficacy, suggesting their potential in diverse medical applications [101]. Semiconductor quantum dots (QDs), owing to their photothermal properties, are also attractive, mainly in the NIR‐II window, enabling deeper tissue penetration in comparison to NIR‐I range [105, 106]. Recent research by Cao et al. displayed the potential of vanadium carbide (V_2_C) QDs as photothermal agents (PTAs) for low‐temperature nucleus‐targeted photothermal therapy (PTT) in the region of NIR‐II [107]. By modifying PEGylated V_2_C QDs with cell nucleus‐targeting TAT peptides and encapsulating them into exosomes functionalized with Arg‐Gly‐Asp (RGD) peptides, they achieved effective cell necrosis at low temperatures upon laser irradiation. Other types of QDs, for instance Si QDs and gold‐carbon QDs, have been loaded onto the outer membrane of exosomes to enable high‐resolution imaging of live cells and target tumor cell metastasis with minimal cytotoxicity [108, 109]. Nevertheless, the large size of QDs poses challenges for encapsulation within exosomes [110]. To overcome this limitation, a DNA hinge ligament has been employed to bind QDs to the surface of exosomes, enabling optimal fluorescent emission while facilitating cellular uptake and visualization of exosome‐QD complexes in tumor cells. This innovative strategy of labeling exosomes with DNA‐bound QDs offers a biocompatible approach for achieving efficient delivery and fluorescence imaging benefits, highlighting the potential of exosome‐QD complexes in biomedical applications. Similarly, mesoporous silica nanostructures (MSNs) are structured materials ideal for targeted drug delivery due to stability and biocompatibility [111]. Recent innovations include exosome‐coated MSNs, promising in tissue engineering and therapeutics. Yong and his research team fabricated tumor exosome‐coated luminescent porous silicon nanoparticles (PSiNPs) for chemotherapy [1]. PSiNPs, synthesized via electrochemical etching, were coated with exosomes (E‐PSiNPs), demonstrating efficient drug uptake and cytotoxicity against cancer cells (Figure 4). A biomimetic nanoparticle (ID@E‐MSNs) using tumor cell‐derived exosomes and porous Si nanoparticles delivered indocyanine green (ICG) and doxorubicin (DOX), enhancing tumor cell uptake and therapeutic effects [112]. In vivo studies confirmed ID@E‐MSNs' efficacy in inhibiting tumor growth and metastasis, showcasing their potential in cancer treatment. Thus, the combination of EVs and nanostructures represents a powerful and versatile approach to overcoming the limitations of current drug delivery systems. These hybrid platforms offer precision, targeting specificity, and reduced side effects, suggesting significant potential for revolutionizing personalized medicine and cancer treatment. Nevertheless, challenges like selecting specific EVs for coating, ensuring efficient drug encapsulation, and maintaining stability in bodily fluids need to be addressed before these technologies can be fully integrated into clinical practice.

### 3.3 Isolation and Separation

The primary challenge in utilizing EVs for clinical applications is the efficient isolation of EVs from small sample volumes within a short time frame. Current methods, for instance ultrafiltration (UF), ultracentrifugation (UC), size exclusion chromatography (SEC), density gradient centrifugation (DGC), and polymer‐based precipitation (PBP), depend on physical properties of EVs such as size, density, and solubility [113], but are often unsuitable for clinical diagnosis [19]. For instance, UF is prone to clogging and can rupture EVs due to applied pressure. UC, a widely used technique, separates particles at high speeds (≈100 000× *g*) but is time‐consuming and impractical for small sample volumes [114]. DGC, which separates particles based on their density, yields higher purity but demands substantial time, biofluid volume, and technical expertise [115]. PBP is effective for low‐input samples but has been associated with contamination [116]. While SEC separates particles by size, its purity is lower than that of UC or DGC [117]. Combining methods such as UC with DGC or SEC with UC/DGC can enhance both efficiency and purity. However, these combinations are often impractical for routine clinical use [118, 119]. Recently, advancements in nanotechnology have paved the way for the development of several EVs separation methods. Numerous nanoarchitectures have been designed for advanced EVs capture technologies, offering high affinity for EVs and enhancing the likelihood of contact between EVs and the capture interface [120, 121, 122, 123, 124, 125]. They can be efficiently functionalized with certain ligands, antibodies, or aptamers that selectively bind to surface markers on EVs like tetraspanins (e.g., CD9, CD63, CD81), proteins responsible for transport and fusion (e.g., flotillin, caveolin‐1), heat shock proteins (e.g., Hsp90), phospholipids, or several lipid‐related proteins. This targeted approach significantly improves the capture efficiency of EVs from complex biological fluids, such as blood or urine [126].

**FIGURE 5:**
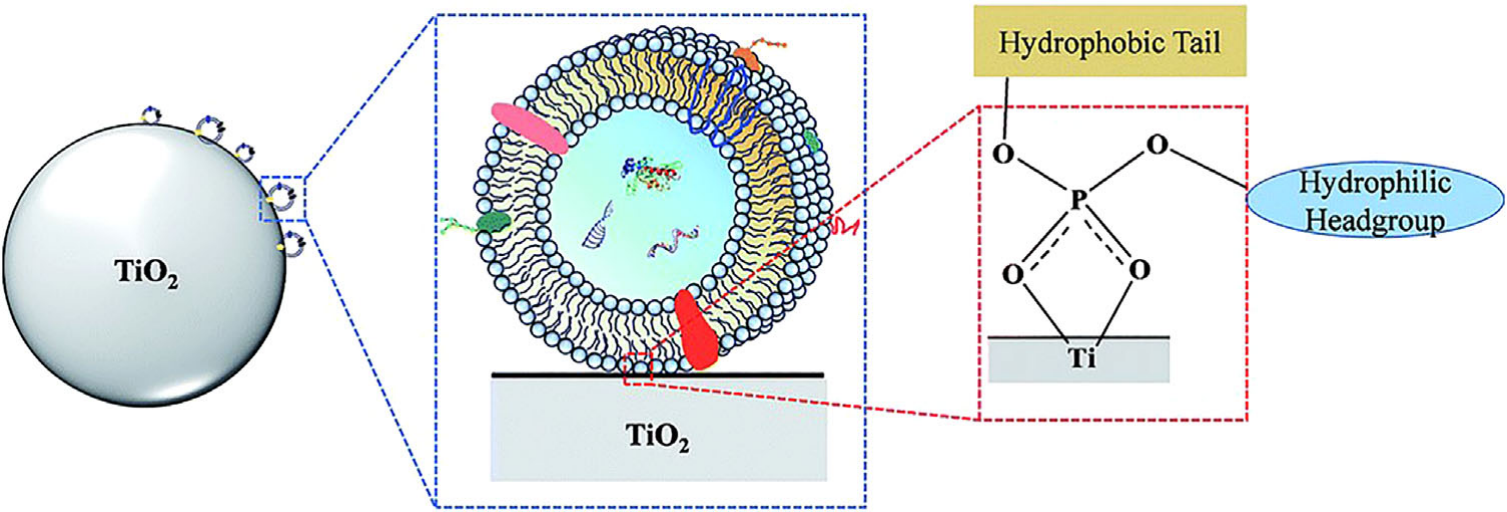
Mechanism for TiO_2_‐based exosome isolation. TiO_2_ particles are utilized for exosome enrichment through bidentate binding between phosphate groups on the lipid‐bilayer surface and TiO_2_. Reproduced with permission from Ref. [133]. Copyright 2019, Royal Society of Chemistry.

Chemical affinity‐based EV isolation can be categorized into two categories, namely nontargeted and targeted capture. Nontargeted capture uses lipid probes, phosphatidylserine affinity molecules, and TiO_2_, whereas targeted capture uses antibodies, aptamers, and peptides. Lipid molecules located on EV surfaces are crucial anchors in affinity‐based isolation. In 2017, Zheng's research group devised a lipid nanoprobe system capable of labeling EVs for enrichment [127]. The system includes 1,2‐distearoyl‐*sn*glycero‐3‐phosphorylethanolamine (DSPE), PEG, and biotin, with an optimized cholesterol‐PEG_1000_‐biotin probe [127, 128]. This approach minimizes damage to EVs and can be combined with functional assays. The outer membranes of EVs are enriched in cone‐shaped lipids such as phosphatidylserine, which have been utilized in EV capture due to their affinity. Annexin A5, a common cellular protein observed in annexin family, has been utilized for cell apoptosis detection and improved EVs isolation efficiency [129, 130]. T‐cell membrane protein 4 (TIM‐4) is categorized as a natural receptor for PS molecules, leading to the development of a TIM‐4‐based EV capturing process [131]. For potential EVs isolation research, other PS‐philic compounds, like MFGE8, have also been investigated [132]. Moreover, the hydrophilic surface of TiO_2_ can bind efficiently to phosphate groups on EV surfaces, making them potential targets for EV capture. In 2019, Gao and the team used micron‐sized TiO_2_ particles to enrich exosomes through bidentate binding between the phosphate groups on the lipid bilayer surface and TiO_2_ (Figure 5) [133]. This coordinate‐based selective enrichment offers several advantages, including enhanced isolation efficiency and minimized nonspecific adsorption, as well as reduced sample processing times due to its simplicity and strong binding affinity. Antibodies targeting EVs biomarkers, including CD9, CD63, and CD81 and cancer‐specific membrane proteins, namely EpCAM, EGFR, and GPC‐1, are other popular tools for EVs capture and isolation [134, 135, 136]. Additionally, aptamers, an effective substitute for antibodies for protein targeting, are bound to antigens such as CD63, EpCAM, PTK7, PSMA, PDGF, and HER2, and have been utilized in EVs isolation due to their lower dissociation constant and reduced risk of triggering undesirable immune responses compared to antibodies [137, 138, 139]. Similarly, peptides comprising a specific affinity for proteins on the surfaces of EVs have also been employed in EVs isolation, including a peptide ligand for EGFR and tumor antigenic peptides for capturing MHC‐I+ EVs [140].

Although chemical affinity‐based EV isolation strategies have significantly advanced EV‐based liquid biopsies, there are still significant challenges to making these techniques clinically viable. Incorporating nanostructures in EV isolation not only enhances reaction efficiency but also addresses issues like interfacial EVs binding, microscale mass transfer limitations, and boundary effects. Thus, combining nanostructures with chemical affinity represents a novel paradigm for highly efficient EV isolation. Nanowires, nanowire‐on‐micropillars, porous structures, wrinkled structures, stacked nanospheres, and other on‐demand structures are highly efficient examples of the structural designs of micro/nanoscale interfacial materials that have been created for EV capture. Graphene‐based materials are used for wrinkled nanostructures [141, 142], while a porous silicon nanowire‐on‐micropillar structure is used for effective trapping of EVs by interstitial locations between the ciliated micropillars [143]. Similarly, a PDMS substrate embedded with silicon nanowires can be utilized for electrostatic collection of urinary EVs. Nanointerfaces can be constructed from nanostructured SiO_2_, and synthetic materials like poly(amidoamine) dendrimers can provide sufficient steric contact with EVs [125, 138, 144].

Physical property‐based EV isolation relies on size distribution, leading to various isolation strategies [145]. The immunoaffinity method uses distinct antibodies to capture EVs, allowing for their isolation in microfluidic devices. For example, Chen and his research group designed a microfluidic immunoaffinity approach to separate EVs from serum samples of glioblastoma patients, providing genetic information about the corresponding tumors [146]. RNA extracted from these EVs offered insights into the genetic profile of the analogous tumor. Reategui and colleagues developed an immunoaffinity‐based herringbone microfluidic device featuring a thermally sensitive nanoarchitectured substrate, enhancing the capture specificity of tumor‐derived EVs from serum and plasma of glioblastoma patients [147]. Using the EVHB‐Chip, they accomplished 94% specificity for tumor EVs with an LoD of 100 EVs/µL. In a separate study, Ko and the team introduced a microchip comprising an ion track‐etched polycarbonate membrane containing pores of 600 nm diameter treated with a soft magnetic film (Ni_80_Fe_20_) [148]. They utilized this strategy to isolate brain‐derived circulating EVs labeled with immunomagnetic nanostructures and utilized it to differentiate specific states of TBI by quantifying miRNA biomarkers encapsulated within these EVs. While this immunoaffinity approach offers high specificity, especially for molecularly defined subtypes, challenges involve the availability of high‐affinity antibodies and the associated high cost. In filtration‐based methods, EVs are isolated based on size using nanoporous membranes or nanowires as filters. Serial filtration is identified as the most widespread approach, however, channel blockage due to similarly sized impurities and fluid shear force hinders its extensive usage [149, 150]. Liang and colleagues engineered a combined double‐filtration microfluidic device followed by a microchip ELISA, attaining a sensitivity of 81.3% along with a specificity of 90% for diagnosing bladder cancer [151]. Despite its fast processing and high throughput, this method lacks specificity and may face issues like filter blockage.

**FIGURE 6:**
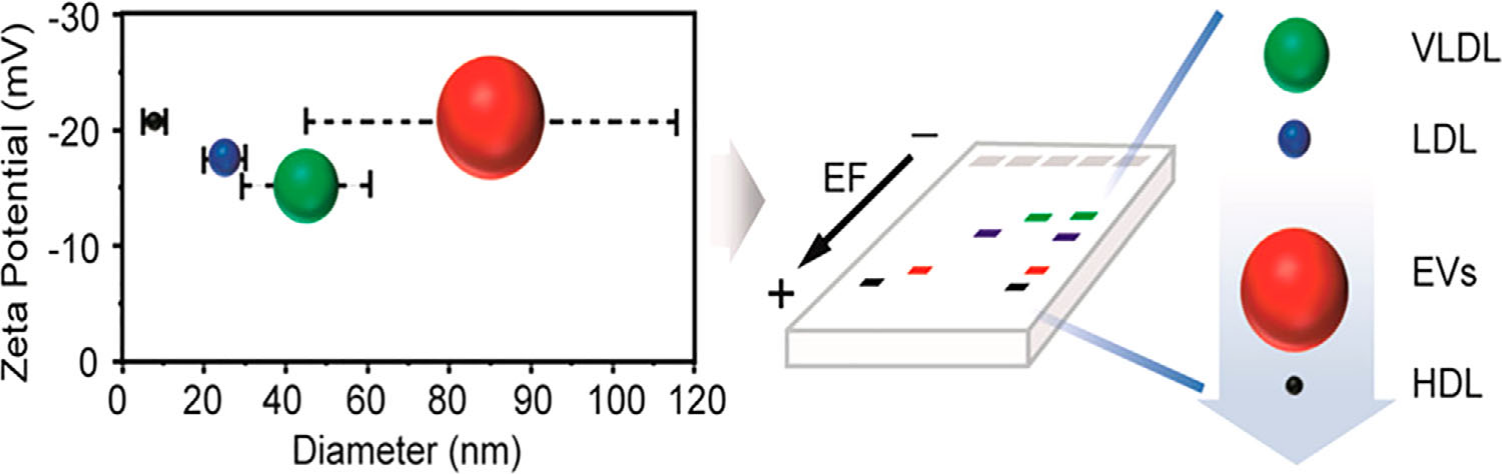
Schematic diagram of sizes and zeta potentials of EVs and lipoproteins. The diagram illustrates the overlap between HDL, LDL, and VLDL in size and zeta potential with EVs, and the isolation of EVs from lipoproteins using agarose electrophoresis. Reproduced with permission from Ref. [161]. Copyright 2020, American Chemical Society.

Moreover, EVs can also be distinguished depending on their hydrodynamic characteristics using asymmetric flow field‐flow fractionation (AF4) technology [152]. Although AF4 can separate soluble particles at the nanoscale based on their density and hydrodynamic characteristics, its use in biomedical research has been restricted due to the devices' complex construction and operation [153, 154]. Other hydrodynamic separation techniques include deterministic lateral displacement (DLD) or viscoelastic flow [155, 156]. DLD is a conventional method that uses pillar arrays to separate microscale particles. It has been used to isolate and separate EVs, integrating into microfluidic platforms [155, 157]. While viscoelastic flow enables good EV purity and recovery with a straightforward chip design, DLD offers a rapid and high‐resolution alternative for EV separation. For example, Wunsch and colleagues employed manufacturable silicon processes to create nano‐DLD pillar arrays, achieving high‐resolution separation of EVs ranging between 20 nm and 110 nm [157]. However, other EV characteristics like density and stiffness can interfere with DLD‐based EV isolation [158]. Electrokinetic techniques like electrophoresis, electromigration, and electroosmosis are used for EV isolation in microfluidics [159, 160]. Electrophoresis is a method that shows distinctive migration rates under an electric field according to charge‐to‐size ratios (Figure 6) [161]. Similarly, Marangoni flow, a contact‐free method, generates a surface tension gradient on the liquid surface, resulting in recirculation flow [162, 163]. These methods provide rapid and cost‐effective EV separation from small sample volumes but may affect EV structure or content and require additional instruments for generating electric fields. Dielectrophoretic (DEP) force, which incorporates electrochemical technology, allows nanoparticles to mov in an asymmetrical electric field. In 2017, Heller's research group developed a technique to isolate EV by means of an alternating current electrokinetic microarray chip using DEP separation force [160]. Other groups have used PDMS‐based microfluidic designs and tools. In 2019, Moore's team demonstrated selective microfluidic fortification of EVs with various antigens [164]. However, DEP's potential biological effects could influence downstream analysis.

**FIGURE 7:**
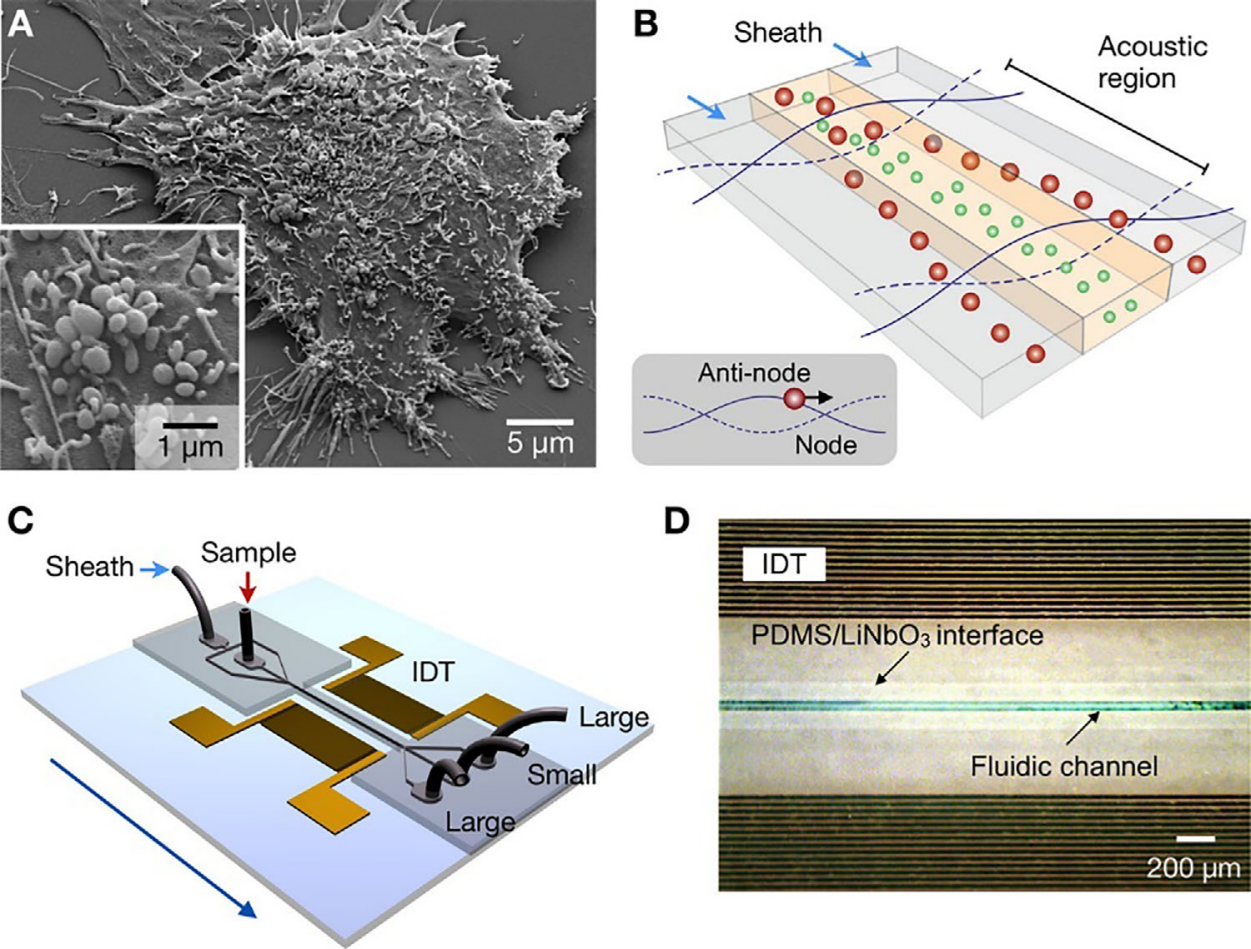
Acoustic nanofilter for microvesicles (MVs) label‐free separation. (A) SEM image of MVs produced by human brain tumor cells (GBM20/3). (B) Schematic of filter operation. MVs in the acoustic region are moved to nodes of the acoustic field section while under acoustic radiation pressure (inset). Since the acoustic force is related to the MV volume, larger MVs travel faster and they get eliminated by sheath flows that are positioned in the node region, whereas small MVs are retained by the center flow. (C) Schematic of the device. An acoustic wave is generated across the flow direction employing two interdigitated transducers (IDT) electrodes. Smaller MVs are gathered at the middle outlet, while large MVs are gathered at the two side outlets. (D) Micrographs showing a working prototype. A piezoelectric (LiNbO_3_) substrate served as the substrate on which the IDT electrodes were patterned. The substrate and the fluidic channel were irreversibly fused. Reproduced with permission from Ref. [166]. Copyright 2015, American Chemical Society.

Surface acoustic waves (SAW) are elastic mechanical waves that can be exploited to isolate EV depending on their mechanical properties. The method could be further modified to isolate EVs under 200 nm with over 99.999% removal of blood cells in whole blood (Figure 7) [165, 166]. SAW‐based acoustic nanofilters offer a contact‐free, size‐tunable approach for EV isolation; however, they cannot separate heterogeneous/coarse cell debris as well as protein aggregates. Thermophoresis is a liquid‐phase‐based isolation technique in which nanoparticles move via a temperature gradient, from high to low temperature regions because collisions with solvent molecules occur in high‐temperature regions. This method has been used to profile surface biomarkers and detect miRNAs inside EVs [167, 168]. Furthermore, thermophoresis‐based EVs‐derived PD‐L1 detection provides suggestive data for early cancer diagnosis and immunotherapeutics prediction [169, 170]. Additional liquid phase‐based detection strategies, including artificial Vir‐FV and droplet digital ExoELISA chips, can detect EVs in the liquid phase, providing a homogeneous basis for EVs counting and biomarker studies, making it a promising approach for EV isolation [139, 171]. Overall, the field is transitioning from purely physical separation toward multifunctional, nanoengineered, and hybrid isolation systems that combine affinity recognition with controlled nanoscale interfaces. The key future requirement is not merely higher capture efficiency, but the development of standardized, clinically robust platforms that balance purity, scalability, cost, and automation. Without addressing these translation barriers, even the most sophisticated nanostructured EV isolation technologies will remain largely confined to research environments rather than becoming routine tools in precision diagnostics.

### 3.4 Detection

Emerging as significant intercellular communication mediators, EVs are involved in a number of physiological and pathological processes, such as immunological responses, cancer, and neurodegenerative disorders. Detection and analysis of EVs hold significant promise for disease diagnosis, prognosis, monitoring, and understanding their biological functions. However, EVs' small size, heterogeneity, and low abundance present challenges for their accurate detection and characterization [172]. The three main categories of traditional EV detection techniques are quantification, visualization, and biochemical testing [113]. Performance of quantification techniques, such as tunable resistive pulse sensing (TRPS), dynamic light scattering (DLS), and nanoparticle tracking analysis (NTA), is mostly reliant on the effectiveness and purity of these isolation methods [173]. However, artifacts introduced during sample handling might impact the outcomes of imaging techniques such as scanning electron microscopy (SEM), transmission electron microscopy (TEM), and cryo‐electron microscopy (cryo‐EM) [3]. Biochemical measurement assays, mainly Western blotting, PCR, and ELISA, are highly specific but are hindered by the heterogeneity of EVs, where nonspecific signals can obscure disease‐specific biomarkers [174]. Additionally, clinical factors like age and sex further complicate detection and analysis. Although guidelines like MISEV2018 have improved the standardization of EV research, the complexity of evolving technologies continues to introduce new challenges [175]. Nanoarchitectonics has significantly contributed to advancing EV detection methods by providing innovative strategies for sensitive, specific, and multiplexed detection. Advanced sensors and single EV detection have decreased the LoD for EVs, addressing their heterogeneity and the crucial needs for sensitive detection technologies. These innovations have facilitated the development of label‐free, rapid, and sensitive detection techniques for EVs, enabling their applications in biomedical research, clinical diagnostics, and therapeutics.

As one approach, optical sensors are used in EV detection which include fluorescent and spectral signals. Linking probes to aptamers or antibodies allows the detection of fluorescent signals [113]. Antibody‐conjugated nanostructures, including AuNPs, QDs, upconversion nanoparticles, graphene oxide (GO), and Ti_3_C_2_ nanosheets, have enabled selective fluorescence signal generation by binding to EVs surface markers [176, 177, 178]. These advancements have made it possible to profile the surface proteins of EVs and quantify them using improved fluorescence signals. Numerous strategies for generating fluorescence signals have been adopted to improve the LoD of EVs. Fluorescence quenching is a method for detecting EVs more sensitively than direct imaging. GO was among the first 2D material identified with fluorescence quenching capabilities, as it binds to aptamers on its surface through strong π–π stacking interactions, where fluorescence is quenched via fluorescence resonance energy transfer (FRET) between dyes and GO [137]. This allows EVs with specific surface proteins to compete and display fluorescent signals [179]. Fluorometric nanosensors have also been developed for this purpose using MoS_2_‐incorporated MWCNTs, quenching phycoerythrin‐conjugated CD63 antibodies, achieving a LoD of 1480 EVs/µL [180]. In addition, nucleic acid base pairing also has the capability to control fluorescent signals. When it attaches to a complementary sequence, such as EVs‐derived miRNA, a molecular beacon, a hairpin‐shaped ss‐DNA with a quenched fluorophore can restore fluorescence [181]. Researchers have designed molecular beacons for simultaneous detection of miRNAs and surface proteins, such as virus‐mimicking fusogenic vesicles [133, 181]. Likewise, spectral signal sensing, including NIR, SPR, and SERS, is also employed to enhance the LoD of EVs. Nanostructures possessing inherent optical properties or catalytic effects, such as AuNPs or Fe_3_O_4_ nanoparticles, have been utilized for colorimetric EV detection. For example, Chen's group developed a visual and label‐free method for detecting tumor‐derived EVs using Fe_3_O_4_ nanoparticles, achieving a low LoD of 3.58 × 10^6^ particles/mL through catalyzing the color change of tetramethylbenzidine [4].

Research on metal nanomaterials has led to a significant decline in the cost of SPR biosensors. Through dual AuNPs signal amplification, the LoD for exosomes was lowered to 5 × 10^3^ particles/mL [182]. Additionally, SPR biosensors constructed on titanium nitride (TiN) nanofilm have demonstrated low LoDs for specific biomarkers in glioma exosomes, CD63 and EGFRvIII, offering potential for monitoring tumor progression [183]. With their amplified and stable Raman signals, metal nanostructures‐particularly nanoparticles, nanorods, nanopillars, and films—facilitate the use of surface‐enhanced Raman scattering (SERS) to detect EVs proteins or RNAs [26, 184]. SPR and SERS techniques have proven successful in detecting EVs biomarkers due to their rapid response, enhanced sensitivity, and specificity. Park and colleagues demonstrated a detection threshold of 10^6^ particles/µL for EVs by employing AuNPs as a substrate to produce SERS signals [185]. Metal‐enhanced fluorescence (MEF) is another advanced optical sensing method for detecting EVs. MEF uses an antibody‐conjugated magneto‐plasmonic nanorod to capture EVs, allowing the release of miRNAs and detecting them by molecular beacons binding to the nanorods [186]. This fluorescent signal could be further amplified by the use of Au plasmons. Similarly, QDs have unique optical properties, including broad and intense absorption and flexible excitation, allowing for greater fluorescence quantum yield. They have been applied to the analysis of EVs‐derived miRNAs and the identification of EVs biomarkers linked to pancreatic cancer, including EpCAM and EphA2 [187]. Self‐powered TiO_2_@MoS_2_ QD‐based probe is capable of ultrasensitive analysis of HOTTIP RNA derived from EVs, accomplishing a detection limit of 5 fg/mL [188]. QDs can be easily enhanced, and their combination with photonic crystals could lead to diagnostic biochips for EVs detection [189]. As spectral signals offer a reduced background and superior LoD compared to fluorescence, this property can be applied for accurate identification of circulating EVs for cancer diagnosis. For instance, by adding quencher‐tagged aptamers to an NIR semiconducting polyelectrolyte complex, Lyu's group created a NIR afterglow luminous nanosensor. When the targeted EVs bind to the aptamers, the distance between the polyelectrolyte complex and the quencher increases, activating the afterglow signal [189]. Another sensitive label‐free technique for multiplex detection of EVs produced from lung cancer cells is SPR signal detection, which senses molecular interactions on Ag/Au surfaces [190]. The design can be further modified to achieve a higher sensitivity using dual‐AuNPs or Au nanocluster membrane‐EVs‐Au nanorod complex [182, 191].

Moreover, electrochemical sensors have benefited from nanomaterial probes such as Ti_3_C_2_ nanosheets, SiO_2_ nanoparticles, and CuS‐enclosed microgels [192, 193]. For example, Sun's research team developed an electrochemical biosensor utilizing Zr‐based MOFs (Zr‐MOFs) for detecting glioblastoma multiforme (GBM)‐derived exosomes, achieving a LoD of 7.83 × 10^3^ particles/µL [194]. This newly invented biosensor had the capability of quantitative and qualitative analysis of exosomes in human serum samples, which were EGFRvIII‐positive, allowing for the differentiation between patients with GBM and healthy individuals. Electrochemical luminescence and colorimetry based on horseradish peroxidase are commonly employed for EV detection on a 3,3′′,5,5′′‐tetramethylbenzidine substrate [195]. Ultrasensitive nanoprobes like ferrocene, Ti_3_C_2_ MXene, and metal ions can improve performance. Dual signal detection systems like nanointerdigitated electrodes and CG‐quadruplex/Hemin, and aptamer‐conjugated Ti_3_C_2_ MXenes have been explored for EVs detection due to their increased surface area and catalytic activity [193]. However, an enhanced LoD of 30 EVs/µL was achieved using AuNP‐enhanced Ti_3_C_2_ MXenes for EVs detection [196]. Moreover, a methylene blue (MB)‐labeled aptamer has been used in ultrasensitive HRP nanoprobes for EVs detection, with a LoD of 0.1 EVs/µL achieved by doping ferrocene onto a ZIF‐67/ITO film and immobilizing black phosphorus nanosheets [197]. Likewise, A range of metal ions with HRP activity, such as Cu^2+^, Fe^3+^, and Ru(bpy)_3_^2+^ ions, have been employed as electrochemical sensors for EVs detection. Techniques include antibody‐conjugated CuS‐enclosed microgels [27], aptamer‐capped Fe_3_O_4_ NPs [4], and Au‐loaded Fe_3_O_4_ nanocubes [6]. Another highly selective and sensitive strategy for cancer‐derived EV detection is the hemin/G‐quadruplex DNAzyme‐based electrochemical aptasensor developed by Li’s group. The platform comprises an anti‐CD63 antibody‐modified gold electrode and a gastric cancer exosome‐specific aptamer linked to a primer sequence complementary to a circular G‐quadruplex template. Target exosome binding triggers rolling‐circle amplification, generating multiple G‐quadruplex units that bind hemin to form horseradish peroxidase‐mimicking DNAzymes. These DNAzymes catalyse H2O2 reduction and generate an amplified electrochemical signal. Through this dual‐amplification strategy, the aptasensor achieved a detection limit of 0.954 EVs µL‐1, demonstrating its potential for the sensitive detection and early diagnosis of gastric cancer [198].

**FIGURE 8:**
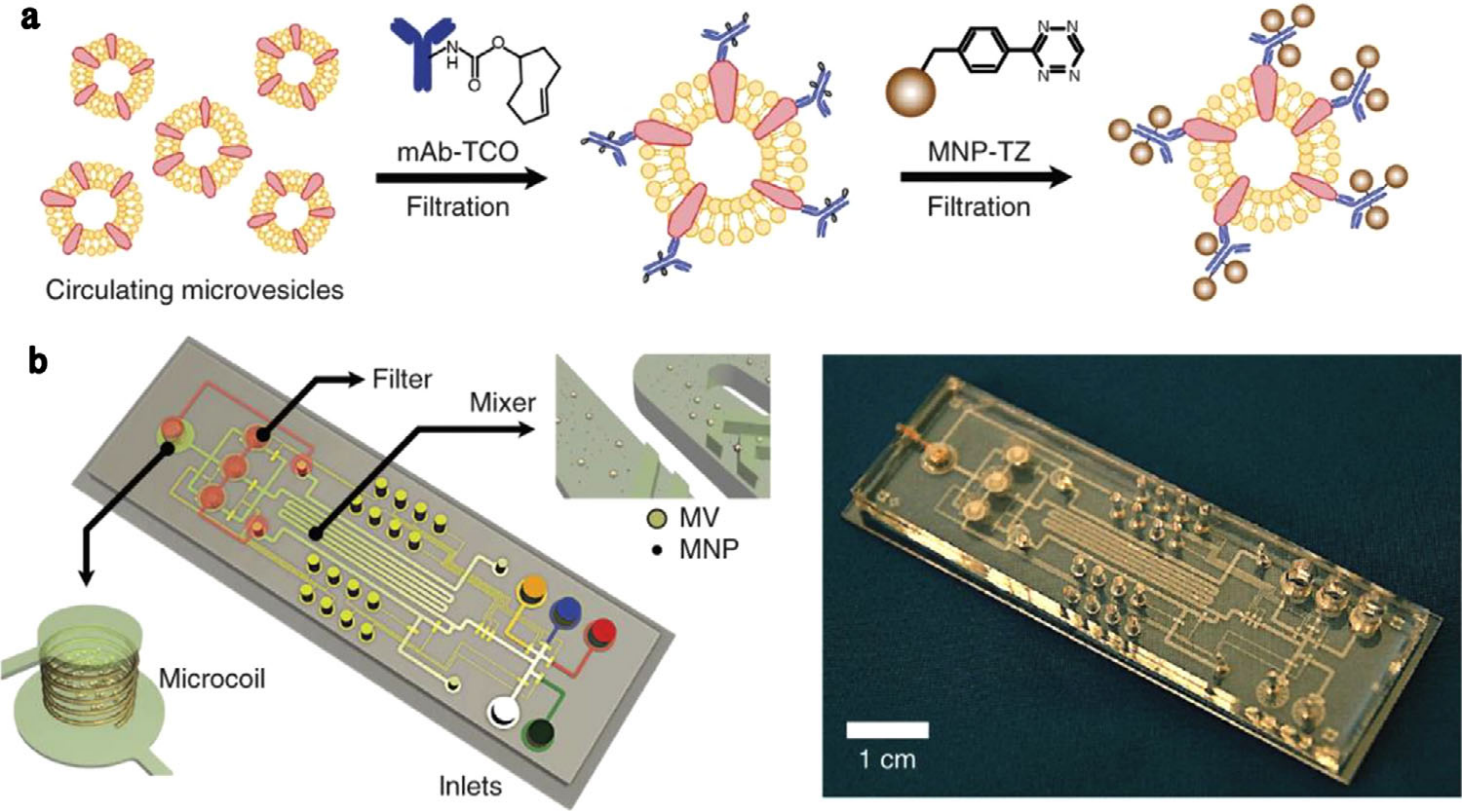
Investigation conducted by Shao involving EVs labeling with target‐specific magnetic nanoparticles (MNPs) on a separate microfluidic chip and profiling of human glioblastoma cells (GBM) biomarkers utilizing a miniaturized micronuclear magnetic resonance (µNMR) arrangement. (a) Schematic of the two‐step labeling procedure employed to optimize MNP binding onto target proteins on microvesicles (MVs). (b) Microfluidic system designed for on‐chip detection of circulating MVs, aimed at detecting MNP‐targeted MVs, concentrating MNP‐tagged MVs (while eliminating unbound MNPs), and enabling in‐line µNMR detection. Reproduced with permission from Ref. [203]. Copyright 2012, Springer Nature.

Apart from optical and electrochemical sensors, several other sensors can also be used to analyze EVs in body fluids, including field‐effect transistor (FET) sensors, magnetic sensors, and mechanical sensors. Electric fields regulate conductivity and transform biological signals into electrical signals, making FET sensors highly sensitive. Two such examples are a reduced graphene oxide (rGO) FET nanosensor coated with AuNP for label‐free plasma EVs quantification and a rGO‐based FET biosensor for EVs detection with a LoD of 20 EVs/µL [199, 200]. FET sensors can also be used to detect miRNAs in EVs [201]. Advanced plasmonic approaches have also further expanded the capabilities of EV analysis. For example, a nanoengineered mesoporous gold (mAu)‐based surface‐enhanced Raman spectroscopy (SERS) platform enabled rapid and ultrasensitive detection of PLAP‐positive EVs in patients with ovarian cancer [202]. In another study, circulating microvesicles were profiled directly from glioblastoma patients' blood samples using a rapid and highly sensitive analytical technique (Figure 8) [203]. A miniaturized nuclear magnetic resonance (µNMR) technology is used to identify MVs after they have been embedded onto a modified microfluidic chip and labeled with magnetic nanoparticles that target particular markers. The system integrates on‐chip micro‐filtration with µNMR techniques to permit quantitative detection of MV amount and protein expression. It performs analysis on small sample volumes, eliminating the requirement for extensive purification or lengthy detection methods. By using a bioorthogonal targeting strategy to selectively bind and densely coat magnetic nanoparticles onto MVs, this technique achieves detection sensitivity that exceeds traditional ELISA and flow cytometry by a number of orders of magnitude. Similarly, electrokinetic and mechanical sensors demonstrate promising results for EV detection offering potential alternatives with unique advantages in sensitivity and specificity [204, 205].

The heterogeneity of EVs presents significant challenges in analyzing them from body fluids. High‐resolution techniques, such as density gradient centrifugation (DGC), have revealed a more confined range of molecules within EVs, and proteins in EVs of different sizes exhibit distinct glycosylation and phosphorylation levels [206]. Single‐cell EV analysis has provided insights into EV characteristics from specific cells, but traditional population measurements still obscure the diversity of EVs discharged from the same cell, highlighting the need for single EV detection. Other detection techniques such as total internal reflection fluorescence (TIRF) microscopy, DNA point accumulation for imaging in nanoscale topography (DNA–PAINT), and advanced platforms like ExoELISA chips have been developed to enable single EV detection [139, 207, 208]. However, multiplex profiling at the single‐EV level continues to be a significant challenge. Innovations like next‐generation sequencing (NGS)‐based platforms and microbead‐assisted flow cytometry (FCM) are opening new possibilities [209, 210, 211]. While capturing detailed information from individual EVs remains challenging, continued advancements in analytical tools hold promise for future breakthroughs in single‐EV detection. Despite major advances in nanoarchitectonics‐enabled optical, electrochemical, and microfluidic sensing, EV detection remains fundamentally limited by biological heterogeneity, low abundance, and lack of standardized analytical workflows. Most current platforms achieve impressive sensitivity in controlled settings, yet struggle with reproducibility, multiplexing at single‐EV resolution, and reliable performance in complex clinical samples.

### 3.5 Therapeutics

Beyond diagnostics, nanoarchitectonics is increasingly transforming EVs into programmable therapeutic nanoplatforms by integrating structural engineering with biological functionality. Through surface functionalization, cargo engineering, biomimetic design, and hybrid nanoengineering, engineered EVs have emerged as highly efficient carriers for nucleic acids, proteins, gene‐editing systems, and small‐molecule therapeutics. Recent advances include bone‐targeted engineered bacterial EVs for miRNA delivery in osteoporosis, exosome‐based targeted delivery systems for restoring osteoblastic bone formation, and engineered exosomes as promising vectors for CRISPR/Cas‐mediated gene‐editing therapies [212, 213, 214]. These studies demonstrate how nanoarchitectonic strategies can precisely regulate EVs composition, targeting capability, and biological function to improve therapeutic efficacy while minimizing off‐target effects.

**FIGURE 9:**
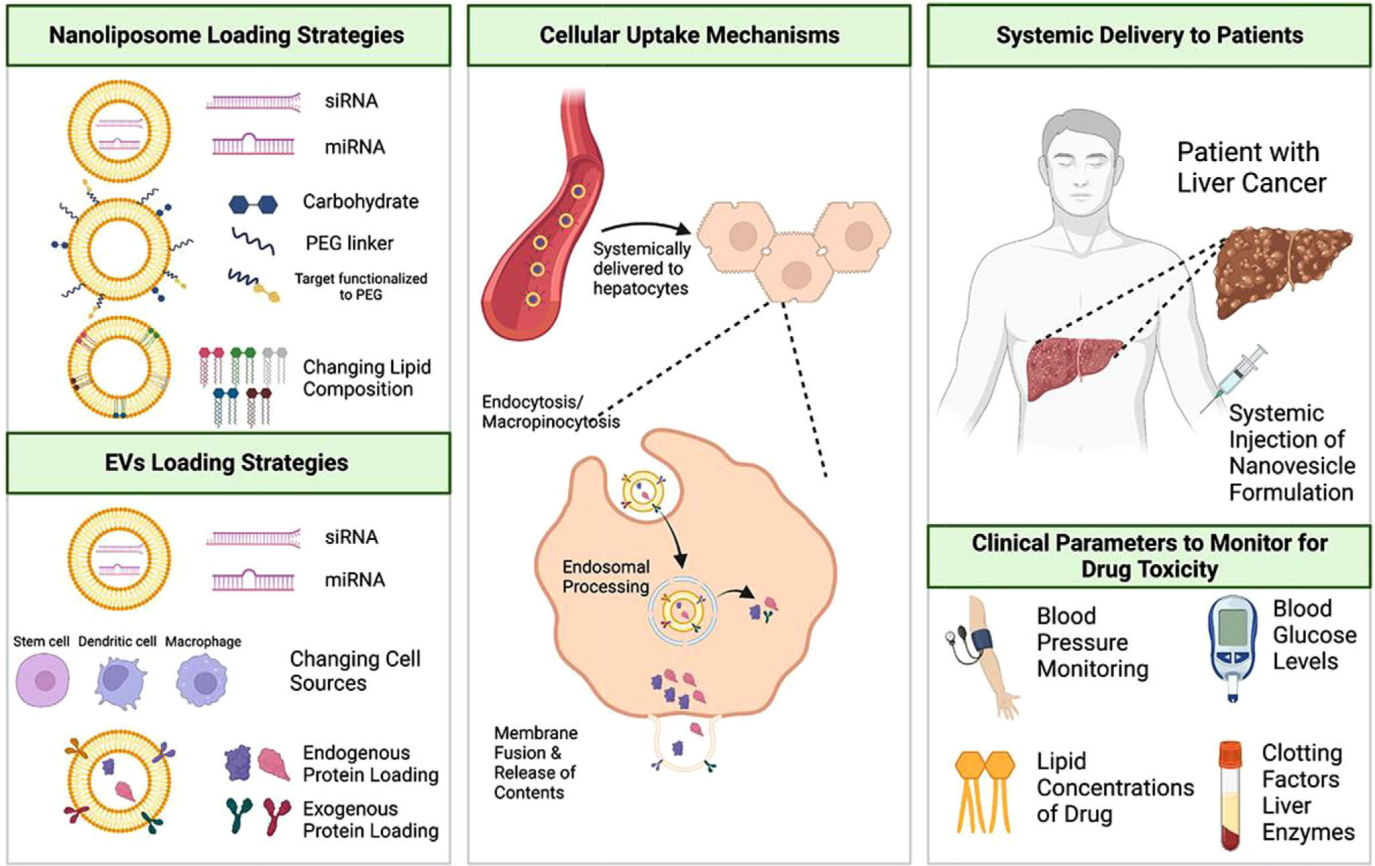
Schematic illustration depicting nanotherapeutics using nanoliposomes and EVs for drug delivery purposes. Additionally, the figure outlines the cellular uptake mechanisms employed by these drug delivery vehicles and highlights clinical parameters crucial for monitoring toxicity in patients. Reproduced with permission from Ref. [217]. Copyright 2024, Taylor & Francis Group, LLC.

By harnessing the unique characteristics of nanostructures, nanoarchitectonics holds immense promise in overcoming key challenges associated with EVs‐based therapeutics, including scalability, stability, targeting efficiency, cargo loading capacity, and controlled release of therapeutic molecules. Importantly, nanoarchitectonics extends beyond conventional delivery design by treating EVs as dynamic nanoscale architectures whose function emerges from hierarchical organization of lipids, proteins, and engineered nanomaterials. From this perspective, EVs performance can be rationally tuned through structural and physicochemical design principles rather than solely through biological modification. A key advancement in this framework is the recognition of EVs membrane mechanics as fundamental nanoarchitectural parameters. Membrane stiffness, elasticity, and curvature critically regulate vesicle stability, circulation behavior, cellular uptake, intracellular trafficking, and cargo release. Membrane stiffness governs EVs deformability and transport across biological barriers, whereas membrane curvature controls protein recruitment, membrane fusion, and vesicle–cell interactions [215]. These interdependent structure–function relationships enable rational nanoarchitectural engineering, where lipid composition, membrane organization, and hybrid nanomaterial integration can be systematically tuned to modulate EVs behavior. Softer EVs generally exhibit enhanced deformability and tissue penetration, while stiffer EVs provide improved structural integrity and cargo protection during systemic circulation. In parallel, curvature‐sensitive design strategies offer opportunities for selective targeting of disease‐associated EVs populations or controlled disruption of pathological vesicular communication. Within this framework, EVs‐based therapeutic nanoarchitectures can be broadly categorized into three complementary strategies: (i) cargo‐engineered EVs systems that enhance loading efficiency and controlled release of therapeutic agents, (ii) membrane‐engineered EV systems that optimize targeting, biodistribution, and biological interactions through modulation of structural and mechanical properties, and (iii) hybrid EVs‐nanomaterial architectures that integrate inorganic or polymeric nanostructures with the intrinsic biocompatibility and biological functionality of EVs. In EVs‐based therapeutics, ensuring biocompatibility, safety, and biodegradability is essential for clinical translation. Nanoarchitectonics enables the design of nanocarriers and scaffolds that minimize immunogenicity and cytotoxicity while maintaining high therapeutic performance (Figure 9). Surface engineering with biocompatible coatings and biomimetic ligands improves stability in biological environments and reduces off‐target effects. Additionally, modular design principles allow incorporation of biodegradable components, enabling controlled degradation and efficient clearance of residual nanomaterials from the body [216].

In EVs‐based therapeutics, ensuring biocompatibility, safety, and biodegradability remains essential for clinical translation. Nanoarchitectonics enables the design of nanocarriers and scaffolds that minimize immunogenicity and cytotoxicity while maintaining high therapeutic performance. Surface engineering with biocompatible coatings and biomimetic ligands improves stability in biological environments and reduces off‐target effects, while modular design allows incorporation of biodegradable components for controlled degradation and safe clearance. For instance, Zhang et al. developed an EVs‐utilized Au nanostructure resembling a popcorn‐like architecture [218]. Initially, EVs served as carriers for doxorubicin (DOX), a commonly used chemotherapeutic drug, followed by self‐assembly of AuNPs around EVs, forming a popcorn‐like nanostructure. The resulting nanopopcorn maintained the photothermal conversion capabilities of AuNP assemblies and the DOX cytotoxicity. Upon exposure to NIR irradiation, the Au nanoplatform generated hyperthermia to ablate tumors and release the drug, enabling combined chemo‐photothermal therapy. This nanoarchitecture showed optimized cellular uptake, controlled drug release, enhanced antitumor efficiency with a tumor inhibition rate of 98.6%, and minimized side effects. Pan et al. reported a non‐invasive method to obtain high‐purity urinary exosomes on a gram scale from patients with gastric cancer [100]. These exosomes were functionalized with multifunctional PMA/Au‐BSA@Ce6 (chlorin e6) nanoparticles using a rapid electroporation approach, forming passion fruit‐like Exosome–PMA/Au‐BSA@Ce6 nanovehicles. These engineered vesicles exhibited efficient tumor cell uptake, prolonged circulation due to immune evasion, and enhanced tumor accumulation. Under acidic tumor microenvironment conditions and 633 nm laser irradiation, the nanoplatform disassembled to release functional components, producing singlet oxygen for effective photodynamic therapy. In vivo studies demonstrated deep tumor penetration and strong therapeutic efficacy in MGC‐803 tumor‐bearing models. Similarly, Niu et al. developed biomimetic EVs‐drug nanostructures by attaching doxorubicin‐loaded heparin‐based nanoparticles onto natural grapefruit‐derived EV surfaces [219]. This strategy significantly increased drug‐loading capacity by approximately four‐fold compared to conventional EVs encapsulation. The engineered EVs‐DNs could cross the blood–brain barrier and blood–brain tumor barrier via receptor‐mediated transcytosis and membrane fusion, enabling efficient delivery into glioma tissues. The system showed prolonged circulation time, enhanced cellular uptake, and strong antiproliferative activity, resulting in superior antiglioma efficacy in vivo. Nanoarchitectonics is also expanding the role of EVs in regenerative medicine through the development of organoid‐derived EVs. Organoid EVs recapitulate tissue‐specific signaling and provide physiologically relevant therapeutic cargos, offering new opportunities for disease modeling and tissue repair. Organoid‐derived EVs have shown considerable promise in bone regeneration by promoting osteogenesis and modulating the bone microenvironment, illustrating the convergence of stem cell biology, organoid technology, and nanoarchitectonics for next‐generation regenerative therapies [214, 220]. However, nanoarchitectonics‐driven EVs therapeutics offer a powerful but still immature platform where cargo loading, targeting, and controlled release can be tuned through rational membrane and hybrid nanostructure design. While membrane mechanics and hybrid EVs‐nanomaterial systems significantly improve delivery efficiency and therapeutic potency, most reported strategies remain constrained to preclinical models with limited translational validation. Critically, the field is still fragmented, with a strong dependence on complex engineering strategies that may compromise scalability, reproducibility, and regulatory acceptance. Future progress should prioritize simplified, modular, and clinically manufacturable EVs nanoarchitectures with well‐defined safety profiles, rather than increasingly complex multi‐component systems that are difficult to standardize for real therapeutic use.

## 4 Nanoarchitectonics: Engineered Nanostructures for EVs Research

Nanoarchitectonics has emerged as a powerful and multidisciplinary strategy for advancing EVs research by integrating concepts from nanotechnology, supramolecular chemistry, materials science, and biotechnology. Rather than focusing solely on the synthesis of nanomaterials, nanoarchitectonics emphasizes the rational design and integration of nanoscale building blocks to construct functional systems with precisely controlled structural and physicochemical properties [221]. This approach encompasses a broad range of processes, including atomic and molecular engineering, organic synthesis, physical material transformation, self‐assembly, external‐field‐directed organization, and nano/microfabrication. Unlike conventional equilibrium‐based self‐assembly, nanoarchitectonics exploits both equilibrium and nonequilibrium processes to generate hierarchical and asymmetric nanostructures with functionalities that are difficult to achieve using traditional material design strategies [222]. The unique advantages of nanoarchitectonics are particularly relevant to EVs research, where conventional analytical platforms continue to face significant challenges arising from the intrinsic heterogeneity, low abundance, and nanoscale dimensions of EVs populations. These limitations often compromise isolation efficiency, molecular specificity, analytical sensitivity, and reproducibility, thereby restricting both mechanistic studies and clinical translation. Nanoarchitectonics addresses these challenges by enabling the rational engineering of nanostructures with tunable morphology, surface chemistry, and physicochemical properties that can selectively interact with EVs, facilitating their efficient capture, enrichment, characterization, and manipulation [221]. Importantly, this design philosophy establishes a direct relationship between material architecture and biological function, allowing nanostructures to be optimized for specific EVs applications rather than relying on universal sensing platforms. Building upon the functional insights discussed in the previous section, this section focuses on how nanoarchitectonics enables the rational design of engineered nanostructures for EVs research. By tailoring material composition, hierarchical architecture, and interfacial properties, nanoarchitectonic platforms can substantially improve target recognition, signal amplification, and molecular accessibility. Consequently, these engineered systems have accelerated the development of highly sensitive biosensors, targeted drug delivery systems, and multifunctional therapeutic platforms for cancer and other diseases [18]. For example, nanoarchitectonics‐based sensing platforms integrating gold nanoparticles and graphene oxide have demonstrated enhanced sensitivity and specificity for detecting cancer‐associated EVs in blood samples, highlighting the potential of rational nanomaterial engineering for early disease diagnosis [18]. Similarly, nanostructures engineered to selectively bind EVs have enabled more efficient loading and targeted delivery of therapeutic agents, improving treatment efficacy while minimizing off‐target effects. Furthermore, nanoarchitectonics‐driven platforms provide valuable tools for investigating fundamental EVs biology, including biogenesis, cargo loading, intercellular communication, and immune modulation, thereby facilitating the discovery of disease mechanisms and novel therapeutic targets [44].

Despite these significant advances, no single nanoarchitectonic platform simultaneously satisfies the full spectrum of requirements for efficient EVs isolation, ultrasensitive detection, multiplexed molecular profiling, scalability, and clinical translation. Instead, each class of nanomaterial leverages distinct optical, electrical, magnetic, catalytic, or porous properties, offering specific analytical advantages while also introducing inherent trade‐offs. As a result, the selection of appropriate nanoarchitecture is highly application‐dependent and requires careful balancing of sensitivity, specificity, stability, throughput, and manufacturability. In response to these limitations, hybrid nanoarchitectures that integrate complementary material properties are increasingly emerging as a rational strategy to enhance overall analytical performance and functional versatility. Importantly, these systems should not be viewed as competing technologies, but rather as synergistic platforms that exploit different transduction and recognition mechanisms to advance EVs analysis. The following subsections critically examine the major classes of nanoarchitectures including plasmonic, fluorescence‐based, magnetic, carbon‐based, and organic framework materials, emphasizing how structural design governs analytical performance, and highlighting their respective strengths, limitations, and evolving roles in clinically translatable EVs technologies.

### 4.1 Plasmonic Nanostructures

Plasmonic nanoarchitectonics is a field focused on engineering nanomaterials with unique optical properties for applications in molecular sensing and diagnostics. The foundation of this technology lies in surface plasmon resonance (SPR), a phenomenon where incident light excites conduction electrons at the interface of materials, triggering resonant oscillations known as surface plasmons. These waves propagate non‐radiatively on the metal–dielectric interface, offering exceptional sensitivity to molecular changes, particularly at the nanoscale. The transition of plasmonic materials to the nanoscale level unlocks a range of possibilities, notably through the utilization of localized surface plasmon resonance (LSPR). The structural properties of plasmonic nanomaterials such as particle size, shape, and surface architecture are critical determinants of their optical behavior and sensitivity. These parameters directly influence field confinement and resonance conditions, thereby allowing precise tuning for effective EV detection and molecular profiling.

**FIGURE 10:**
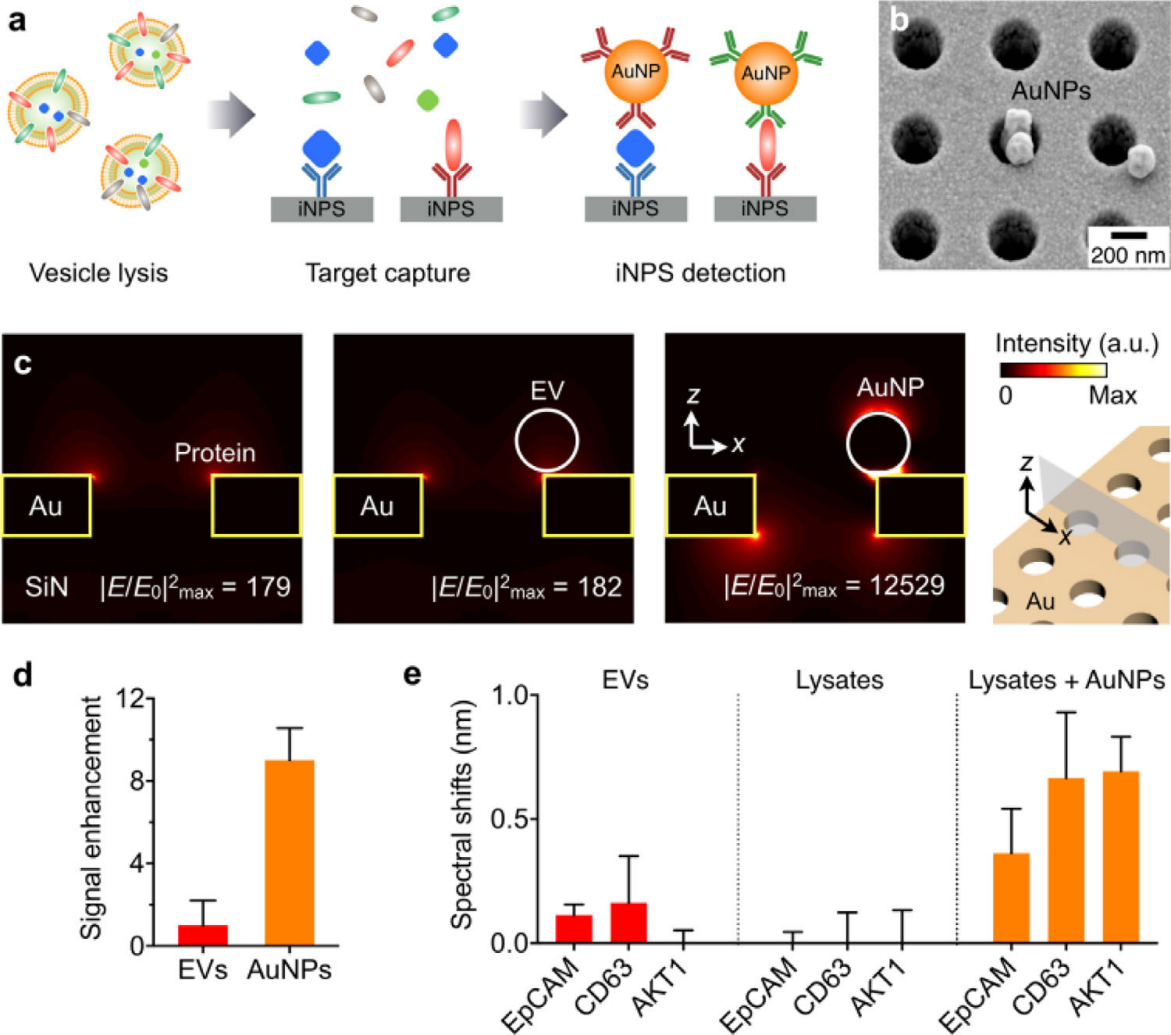
(a) EVs are lysed to discharge molecular cargos, collected on the iNPS chip using affinity ligands, and tagged with AuNPs. The assay accommodates both transmembrane and intravesicular proteins. (b) SEM image displaying AuNPs after iNPS steps. (c) FDTD simulation illustrating AuNPs on the surface of iNPS concentrating electrical fields, enhancing field intensity up to 70‐fold compared to protein or EVs binding. (d) Signal enhancement: Spectral shift is nine‐fold higher with AuNPs (100 nm) on the iNPS chip compared to EVs binding alone. (e) iNPS assay validation: AuNPs enhance detection of membrane proteins (EpCAM, CD63) and intravesicular protein (AKT1) with improved spectral shifts. Reproduced with permission from Ref. [15]. Copyright 2018, American Chemical Society.

Among the plethora of plasmonic nanomaterials, colloidal gold nanoparticles (AuNPs) have emerged as a cornerstone due to their intriguing LSPR‐enhanced optical characteristics [18]. Tailoring the size, shape, and structure of AuNPs enables precise control over their optical response, extending from the visible to the NIR region. The applications of plasmonic nanoarchitectonics in molecular sensing, particularly for the detection and analysis of EVs, have received considerable attention. Traditional techniques such as SPR sensing, utilizing Au‐coated sensor chips, have paved the way for sensitive and rapid detection of EVs [223, 224, 225]. By functionalizing these chips with specific antibodies, researchers can capture and detect EVs based on their surface protein markers, enabling potential applications in the field of cancer diagnosis and monitoring. To further enhance sensitivity and throughput, SPR‐based nano‐plasmonic exosome (nPLEX) sensors have been developed, incorporating periodic nanohole arrays for improved electromagnetic enhancement [226]. The system relied on optical transmission through periodic nanoholes instead of total internal reflection, a process commonly employed in commercial SPR systems. The chip consisted of a lattice of nanoholes on 200‐nm‐thick gold film with detection antibodies attached via a polyethylene glycol (PEG) linker. Exosomes were introduced into the flow cell, and the corresponding spectral shift was calculated to detect target‐specific proteins on the exosomes. The probing depth (<200 nm) of plasmonic nanoholes aligned well with the size of exosomes, thus exhibiting remarkable sensitivity, permitting the detection of EVs at concentrations as low as 10^3^ per mL, surpassing conventional methods by 10^4^ times with Western blot and 10^2^ times with ELISA. Integration with microfluidic platforms further enhances their utility for high‐throughput analysis of multiple samples concurrently. Additionally, the transmission setup facilitates system miniaturization and the creation of densely packed sensing arrays. Intriguingly, advancements such as the intravesicular nanoplasmonic system (iNPS) offer a preview into the future of EVs analysis, enabling the interrogation of both intravesicular and transmembrane proteins on a single device [15]. In particular, the signal is amplified by first capturing the molecular targets in EV lysates on the plasmonic device and then labeling them with AuNPs. This technique offers a unified format for high‐throughput and automated detection, independent of the target protein's position within the EVs. Protein markers in cancer‐derived EVs can be screened using iNPS, which shows that EVs have cytosolic and membrane proteins whose expression levels match those in the parent cells. Furthermore, the research group discovered that drug treatments altered the amount of intravesicular proteins, suggesting that EV analysis could be useful for monitoring treatment responses (Figure 10).

Another SPR‐based sensor employing a self‐assembled monolayer of gold nano‐islands (SAM‐AuNIs) on an Au‐coated glass substrate achieved a LoD of 0.194 µg/mL while enabling discrimination between exosomes and microvesicles across diverse biological samples [227]. Building on this plasmonic amplification strategy, dual AuNP‐supported signal enhancement has been introduced, where aptamer/T30‐functionalized AuNPs are linked to exosome surface proteins, yielding a markedly improved LoD of 5 × 10^3^ exosomes/mL, substantially outperforming conventional ELISA [182]. Extending this concept to spatially resolved formats, AuNP‐enhanced SPR imaging (SPRi) microarrays have enabled multiplexed exosome profiling with a reported LoD of 10^7^ exosomes/mL [190]. Beyond SPR, plasmonic nanostructures have also been integrated into scattering‐based detection. A nanoplasmon‐enhanced scattering (nPES) assay utilizing antibody‐functionalized spherical AuNPs and AuNRs enabled simultaneous detection of exosomal surface markers by exploiting dark‐field scattering signals, achieving sensitivity down to single‐particle levels [228]. In parallel, surface‐enhanced Raman scattering (SERS) platforms have enabled both label‐free profiling of exosomal molecular fingerprints and targeted detection using SERS‐active nanotags, allowing discrimination between healthy and tumor‐derived exosomes. In addition to spectroscopic approaches, plasmonic nanomaterials have been widely adapted into point‐of‐care formats. Lateral flow assays (LFAs) leverage the strong optical absorbance of AuNPs (∼520 nm) to enable rapid colorimetric detection of EVs within minutes, supporting decentralized diagnostic applications [229, 230]. In LFAs, antibody‐functionalized AuNPs serve as recognition and reporting elements for specific exosomal antigens [229, 231, 232]. More recently, engineered AuNP‐based “smart probes” have further expanded colorimetric EVs detection, where target‐induced aggregation produces a visible color shift, while catalytic AuNP activities enable enzyme‐like signal amplification for disease‐relevant biomarkers such as hepatocellular carcinoma [29]. Complementing these optical approaches, electrochemical biosensors incorporating Cu and Ag nanostructures have demonstrated high analytical sensitivity for EVs detection, including applications in prostate cancer diagnostics [233]. In parallel, mechanical transduction strategies such as cantilever arrays and surface acoustic wave (SAW)‐based amplification have broadened plasmonic sensing toward dynamic EVs tracking and in vivo imaging [234]. These plasmonic and plasmon‐coupled nanostructures leverage localized electromagnetic field enhancement and related transduction mechanisms to achieve highly sensitive and, in many cases, multiplexed detection of exosomal biomarkers. Their compatibility with microfluidic integration and portable readout formats further supports their translation toward point‐of‐care diagnostics. In contrast to these primarily optical signal‐amplification strategies, fluorescence‐based nanostructures provide complementary advantages through tunable emission, multicolor encoding, and real‐time visualization of EVs dynamics.

### 4.2 Fluorescent Nanostructures

Fluorescent nanostructures, especially quantum dots (QDs) and upconversion nanoparticles (UCNPs), have revolutionized EVs research by providing sensitive, multiplexed, and versatile detection platforms. These nanostructures are particularly powerful for EVs analysis because their advanced optical design enables high‐sensitivity detection of low‐abundance EVs and their molecular cargo, which is crucial for early disease diagnosis and monitoring therapeutic responses. Importantly, their structural attributes, including size, crystallinity, and surface functionalization critically determine optical output, biocompatibility, and colloidal stability, thereby directly influencing detection performance and targeting efficiency. In addition, their ability to enable real‐time tracking of EVs in living cells and organisms allows systematic investigation of EV dynamics and their roles in intercellular communication. Furthermore, the intrinsic multiplexing capability of QDs and UCNPs supports simultaneous profiling of multiple EVs‐associated biomarkers, thereby providing a more comprehensive understanding of EV molecular composition and functional heterogeneity. Collectively, these innovations in fluorescent nanostructure design are driving significant progress in EVs research, offering powerful analytical tools for diagnostics, therapeutics, and personalized medicine.

**FIGURE 11:**
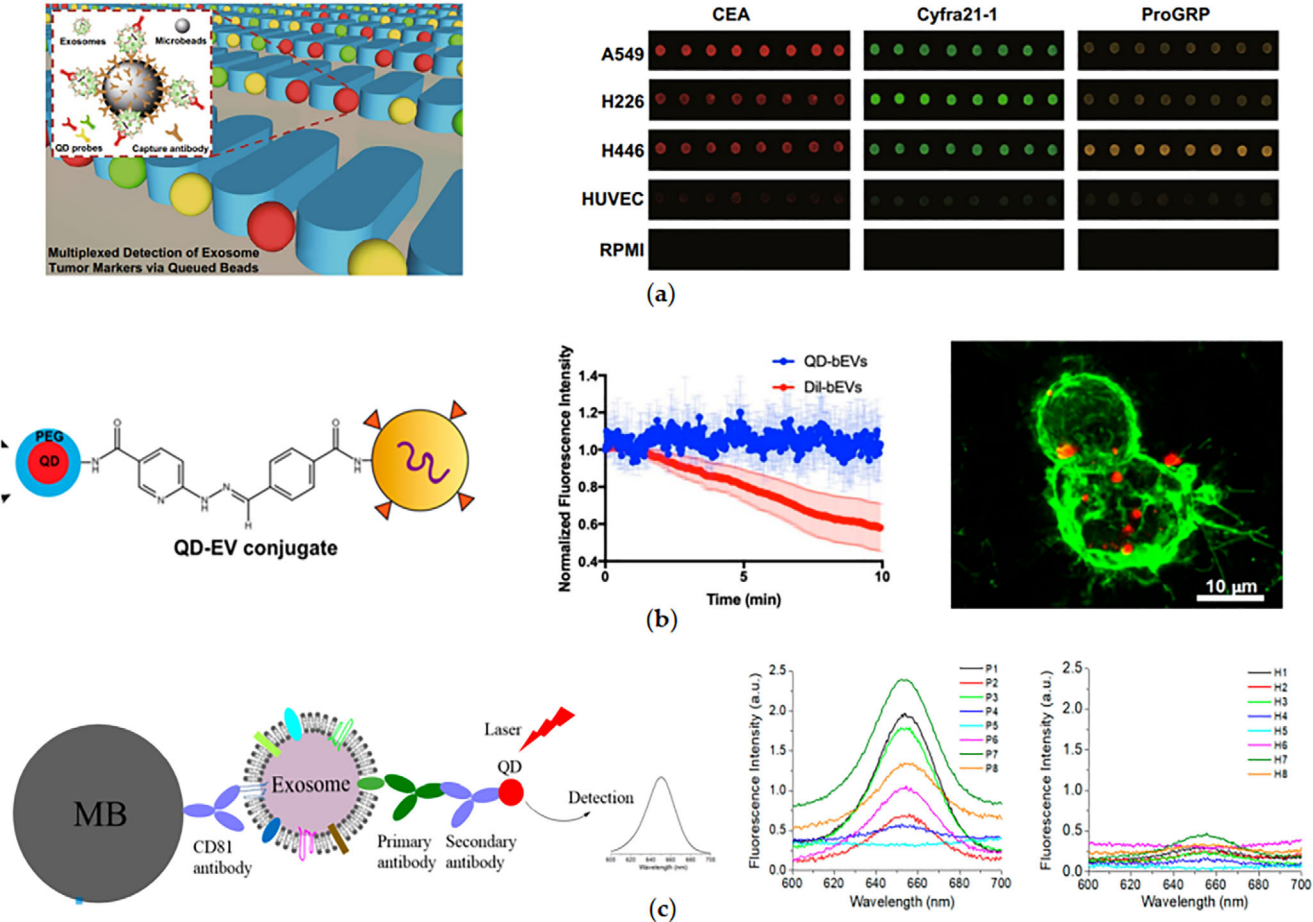
Fluorescent detection of exosomes utilizing QDs. (a) Exosomal tumor markers are isolated and detected in a multiplexed manner employing QDs and magnetic beads within a microarray chip. The schematic depicts exosome separation with single magnetic beads trapped in microfluidic pillars and multiplexed detection with multicolor QDs. Fluorescence images display exosomes from different cell lines labeled with three target‐specific QDs: QD525, QD585, and QD625. Reproduced with permission from Ref. [239]. Copyright 2019, Springer Nature. (b) Exosomes are labeled and visualized using QDs. The schematic illustrates EVs labeling with QDs, showcasing the photostability of QDs compared to traditional DiI (lipophilic) organic dyes. Fluorescence imaging reveals the distribution of QD‐labeled EVs (red) on lectin‐stained BV‐2 cells (green). Reproduced with permission from Ref. [238]. Copyright 2020, American Chemical Society.(c) Breast cancer detection is demonstrated with QDs combined with immunomagnetic separation. The schematic depicts immunomagnetic isolation with magnetic beads and fluorescence detection of surface protein markers by QD655. The fluorescence spectrum of exosomes from HER2‐positive breast cancer patients and healthy donors, labeled with HER2‐targeted QDs, highlights the diagnostic potential of QDs for detecting HER2‐positive breast cancer. Reproduced with permission from Ref. [242]. Copyright 2021, MDPI, Basel, Switzerland.

Among fluorescent nanostructures, QDs represent a class of semiconductor nanocrystals with unique optical properties, making them ideal candidates for EV detection. Owing to their size‐dependent electronic structure, QDs (typically 2–10 nm in diameter) can generate bright multicolor fluorescence under single‐wavelength excitation, due to their narrow emission spectra and broad absorption profiles [235]. In addition, QDs exhibit excellent photostability, enabling prolonged imaging and repeated measurements without significant signal degradation. These attributes, together with their commercial availability and facile conjugation with a range of ligands, make QDs highly efficient fluorescent labels for EV detection through both imaging and spectroscopic approaches [189, 236, 237, 238, 239, 240, 241]. One notable application of QDs in EV research is their utilization in multiplexed detection platforms. In an excellent study, queued beads containing QDs in a microarray configuration were used to multiplex the detection of tumor exosomes (Figure 11a) [239]. After extraction, the exosomes were affixed to magnetic microbeads, which were then confined within micropillars on a microfluidic chip. Fluorescence imaging was then used to identify the presence of QDs attached to the exosomes adhered to the microbeads. The micropillar arrangement facilitated the individual partition of microbeads, ensuring that the fluorescence signals emitted from QDs were evenly distributed across each bead. This setup effectively prevented optical interference, thereby enhancing the accuracy of the test results. By employing QDs of diverse colors and sizes conjugated with specific cancer markers, they achieved simultaneous detection of exosomes derived from various cancer cell lines. This innovative approach enabled the accurate quantification of cancer markers on exosomes, showcasing the potential for clinical diagnosis and monitoring.

Beyond multiplexed sensing, QDs have also been widely applied in live‐cell and tissue imaging of EVs. One study utilized QDs conjugated to exosomes via click chemistry (4FB, 4‐formylbenzoate to HyNic, 6‐hydrazinonicotinate acetone hydrazone) to visualize semen‐derived EVs in human vaginal mucosa and brain‐derived EVs in real time (Figure 11b) [238]. This chemistry is sensitive under mild conditions and can be tailored, leading to high labeling efficiency while preserving the optical properties of QDs and the function of the biological target. This QD‐EV conjugate exhibited remarkable resistance to photobleaching in comparison to EV labeled with DiI, a frequently used EV staining dye. QD‐EV conjugation can be optimized by adjusting the initial QD to EVs ratio and using catalysts to accelerate the hydrazone formation process. More significantly, the QD‐EVs conjugates demonstrated ex vivo fixed‐imaging ability and high‐resolution live‐imaging capability for EVs in vitro. This technique allowed for the colocalization of semen EVs with human vaginal cells in fixed tissue sections as well as the live imaging of the interaction of brain EVs with BV‐2 microglial cell membranes. The study of EV localization and function in cellular and tissue contexts was simplified by this labeling technique, which also yielded valuable insights into disease pathophysiology and intercellular communication.

In addition to imaging‐based applications, QDs have been extensively integrated into quantitative assays for EV surface protein detection. By combining magnetic separation with QD‐based labeling, researchers achieved sensitive detection of exosome surface markers associated with cancer (Figure 11c) [242]. In this approach, magnetic beads functionalized with CD81 antibodies were used to isolate exosomes from cell culture media and human plasma samples, while QD655 indirectly labeled cancer‐associated markers via secondary antibody binding using streptavidin–biotin chemistry. Fluorescence signals were measured using a standard fluorometer, enabling quantitative analysis of exosomal biomarkers. This method demonstrated enhanced sensitivity with a LoD of 4.7 × 10^7^ exosomes/mL and showed strong agreement with ELISA (Pearson correlation coefficient: 0.986). Moreover, differential expression analysis of EpCAM, HER2, CD44, and CD24 across breast cancer cell lines enabled clear discrimination from healthy controls, while clinical validation confirmed HER2‐positive exosomes as a strong diagnostic indicator for HER2‐positive breast cancer (AUC ≈ 0.97). The simplicity of this workflow comprising magnetic isolation, antibody incubation, QD labeling, and fluorometric readout makes it particularly attractive compared with more complex analytical platforms.

Beyond optical and immunoassay‐based strategies, QDs have also been extended into electrochemical and electroluminescent EVs sensing platforms. In 2017, Boriachek et al. developed an electrochemical detection strategy in which CdSe QDs functionalized with anti‐HER2 and anti‐FAM134B antibodies were used to selectively target breast and colon cancer‐derived exosomes [243]. By dissolving the QDs under acidic conditions, they quantified the release of Cd^2+^ ions using anodic stripping voltammetry at a bare glassy carbon working electrode. This approach demonstrated high sensitivity, achieving a LoD of 10^5^ exosomes/mL. Additionally, QDs have been utilized to improve electrochemiluminescence signals (ECL) through self‐assembly with Ru(dcbpy)_3_^2+^ to minimize energy wastage and reduce the distance for electron transfer [244]. Furthermore, MoS_2_ QDs were employed to construct a self‐driven photoelectrochemical biosensor for detecting exosomal RNA, in conjunction with TiO_2_ nanosilks [188]. Beyond QDs, other fluorescent nanostructures, such as upconversion nanoparticles (UCNPs), have also shown promise in EVs detection [178, 245, 246, 247, 248]. For instance, a luminescence resonance energy transfer (LRET) aptasensor was designed that used UCNPs and plasmonic gold nanorods (AuNRs) to detect exosomes [178, 246]. This strategy enables efficient energy transfer modulation and quantitative EVs analysis with high specificity, highlighting the versatility of UCNP‐based systems in biosensing applications. Moreover, recent advances in super‐resolution fluorescence microscopy have enabled single‐EV detection and characterization, where UCNPs can be resolved at the level of individual small EVs, allowing nanoscale mapping of surface antigen distribution and heterogeneity [249]. While fluorescence‐based systems primarily rely on optical signal generation and multiplexed imaging, magnetic nanostructures provide a complementary strategy by enabling efficient EVs isolation, enrichment, and detection through external magnetic manipulation and separation.

### 4.3 Magnetic Nanostructures

Magnetic nanostructures, including magnetic nanoparticles and magnetic nanowires, have emerged as powerful tools in EVs research due to their unique magnetic properties and versatile applications [6]. Their structural characteristics such as core‐shell morphology, surface coating, magnetic core size, and functionalization play a pivotal role in influencing magnetic responsiveness, biocompatibility, and binding efficiency for EVs capture and detection. Rational engineering of these structural parameters is therefore essential to maximize capture efficiency while minimizing nonspecific adsorption and preserving EVs integrity. Consequently, magnetic nanostructures have become one of the most widely employed nanoarchitectures for the separation and purification of EVs from cell culture supernatants and complex biological fluids [250, 251, 252, 253, 254, 255]. By functionalizing MNPs with specific ligands targeting EV surface markers, researchers can selectively capture and concentrate EVs from complex biological matrices [104]. Compared with conventional ultracentrifugation, magnetic separation offers several important advantages, including high isolation efficiency, rapid processing, reduced sample handling, and seamless compatibility with downstream analytical techniques. Nevertheless, the performance of magnetic capture remains highly dependent on the choice of affinity ligand and the heterogeneous expression of EV surface biomarkers, which may introduce isolation bias toward specific EV subpopulations and potentially overlook biologically relevant vesicle subsets.

**FIGURE 12:**
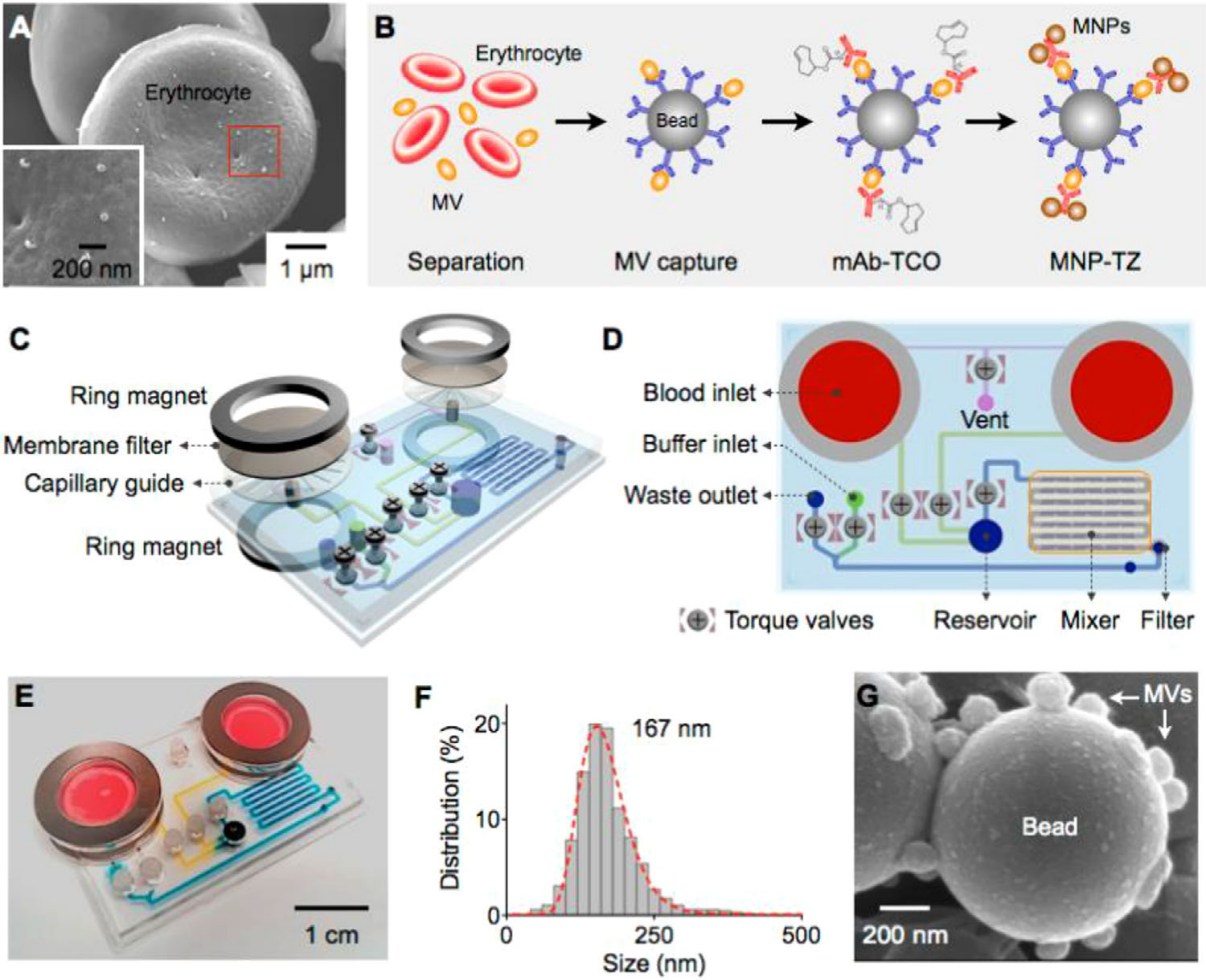
Construction of magnetic nanosensor for the detection and profiling of microvesicles (MVs) derived from red blood cells (RBCs). (A) Scanning electron micrograph of an erythrocyte with budding MVs from a packed RBC unit (pRBC). (B) Assay design scheme: MVs are detached from blood and captured by antibody‐coated microbeads (1 µm in diameter). Immunomagnetic labeling renders the beads superparamagnetic, followed by a cycloaddition reaction between trans‐cyclooctene (TCO) and tetrazine (TZ) to couple magnetic nanoparticles (MNPs) to antibodies (mAbs). (C) Exploded view of the micro‐NMR (µNMR) system, displaying the membrane filter and capillary guide sandwiched between two ring magnets, allowing for easy replacement of the filter set during sample processing. (D) Device components include two inlets for blood loading, torque‐assisted valves for fluidic management, a herringbone mixer, and an embedded filter for microbead capture. MVs are accumulated into the reservoir before other reagents (microbeads, mAb‐TCO, MNP‐TZ) are serially loaded. (E) Photograph of a prototype device measuring 76 × 52 mm^2^, capable of filtering 300 µL (150 µL per inlet) of pRBCs from a single sample load. (F) Size dispersion of filtered MVs analyzed by nanoparticle tracking analysis (NTA), with an average size of 167 nm. (G) Electron microscopy confirms effective capture of MVs by microbeads following labeling with the fabricated microfluidic device. Reproduced with permission from Ref. [259]. Copyright 2013, American Chemical Society.

Beyond EVs enrichment, magnetic nanostructures have enabled the development of integrated analytical platforms capable of combining isolation, labeling, and quantitative detection within a single workflow. Lee and colleagues pioneered the development of microfluidic devices integrated with micro‐nuclear magnetic resonance (µNMR) technology for the isolation and analysis of MNP‐bound EVs [256]. Utilizing the µNMR principle based on transverse decay rate measurement (R2), which correlates with MNP concentration, the method enables quantification of target EVs via MNP labeling [256, 257, 258]. With enhanced sensitivity, it outperformed Western blotting, ELISA, and nanoparticle tracking analysis (NTA) by factors of 10^4^, 10^3^, and 10^2^, respectively. By integrating rapid isolation, a new miniaturized nuclear magnetic resonance (µNMR) system with a membrane filter and capillary guide allows for efficient microvesicle (MV) isolation from blood, MNP labeling, and detection, all on‐chip (Figure 12) [259]. Although the device enriched MVs directly from packed red blood cell (pRBC) units and labeled them with target‐specific MNPs, the platform was able to detect MVs in pRBC units with speed and accuracy. The identification of important molecular markers (CD44, CD47, and CD55) and precise MV quantification were made possible by subsequent detection utilizing a µNMR technique. Importantly, the device enabled longitudinal monitoring of MV accumulation in stored blood units, demonstrating that MV concentration progressively increases during storage and may serve as a reliable indicator of blood aging. The platform also revealed the oxidative stress potential and nitric oxide‐related activities associated with stored blood products. This study highlights how magnetic nanoarchitectures can transform EVs analysis from a labor‐intensive laboratory procedure into an integrated, miniaturized, and clinically translatable diagnostic platform. However, despite their impressive analytical performance, widespread clinical implementation will require further improvements in device standardization, assay reproducibility, throughput, and cost‐effective manufacturing.

Magnetic nanostructures also serve as versatile signal amplification and readout agents for detecting molecular constituents of EVs. Chen et al. demonstrated the use of functionalized Fe_3_O_4_ nanoparticles as nanocarriers of EpCAM‐specific aptamers for the detection of targeted exosomes [4]. Upon binding to exosomes, the aptamer‐conjugated MNPs exhibited altered catalytic activity, enabling quantitative measurement of targeted exosomes based on colorimetric changes. This approach provides a rapid and sensitive method for detecting specific subpopulations of EVs, offering potential applications in disease diagnosis and monitoring. Beyond molecular recognition‐based sensing, magnetic nanostructures also enable integrated EVs enrichment and detection workflows. For example, functionalized superparamagnetic mesoporous flower‐shaped nickel ferrite spheres (SMNFs) were developed as a rapid and highly sensitive sensing platform for pregnancy complication screening through the detection of placental EVs biomarkers. The high surface area and magnetic responsiveness of these nanostructures facilitated efficient EVs capture while enhancing analytical sensitivity for colorimetric detection [260]. Furthermore, magnetic nanostructures have been incorporated into lateral flow immunoassay (LFIA) platforms to achieve rapid and instrument‐free EVs detection [261]. The peroxidase‐like activities of MNPs coated with fatty acids of varying chain lengths (oleic acid, myristic acid, and lauric acid) were assessed in solution using H_2_O_2_ and 3,3,5,5‐tetramethylbenzidine (TMB) and on strips via a biotin‐neutravidin affinity assay. By conjugating Fe_3_O_4_ nanozymes with specific antibodies targeting EVs surface markers, researchers can visualize EVs capture on LFIA strips based on the brown color of the MNPs. Consequently, MNPs coated with oleic acid were utilized as colorimetric labels to detect plasma‐derived EVs in LFIAs due to their nanozyme effects. This simple yet effective detection method offers rapid and point‐of‐care detection of EVs, making it suitable for decentralized diagnostic settings and resource‐limited environments. Despite these advantages, LFIA‐based platforms generally exhibit lower multiplexing capability and quantitative precision than laboratory‐based analytical methods, highlighting the need for further integration with advanced nanomaterials to enhance analytical sensitivity and multiplexed detection.

Superparamagnetic iron oxide nanoparticles (SPIONs) represent a subset of nanostructures recognized for their magnetic properties and high safety profiles [262]. When functionalized with bioactive agents or incorporated into composites, SPIONs exhibit promising theranostic potential for diverse diseases, especially central nervous system disorders [263]. Their remarkable superparamagnetic characteristics allow them to accumulate in specific tissues when exposed to an external magnetic field. By introducing SPIONs, engineered EVs gain improved targeting abilities, facilitating their accumulation in desired tissues via magnetic guidance. For example, EVs filled with SPIONs and curcumin were directed to gliomas using an external magnetic field, eliciting potent antitumor effects [264]. Similarly, Kim et al. developed SPION‐incorporated EVs‐like nanovesicles for systemic treatment of spinal cord injuries, with magnetic field guidance significantly increasing their presence at the injury site [265]. This magnetic navigation approach was also effective in enhancing EVs localization to ischemic lesions in mice with ischemic stroke. Additionally, SPION uptake induced the expression of therapeutic growth factors in donor mesenchymal stem cells (MSCs) and their resultant hybrid EVs [266]. Moreover, these hybrid EVs possess magnetic hyperthermia capabilities due to SPION incorporation, demonstrating a synergistic antitumor effect on glioma cells when combined with curcumin and magnetic hyperthermia [264]. Overall, magnetic nanostructures combine EVs isolation, detection, and guided delivery, but challenges such as capture bias, functionalization variability, and limited multiplexing still need to be addressed. Hybrid systems that pair magnetic enrichment with complementary sensing are therefore promising. In this context, carbon‐based nanostructures offer high conductivity, large surface area, chemical stability, and tunable surface chemistry, making them especially valuable for next‐generation EVs biosensing, as discussed in the following section.

### 4.4 Carbon Nanostructures

Carbon nanostructures, namely carbon nanotubes (CNTs), graphene, and graphitic carbon nitride nanosheets (g‐C_3_N_4_ NSs), have garnered outstanding attention in EVs research due to their unique properties and versatile applications. Unlike magnetic nanostructures, which primarily rely on magnetic responsiveness for EVs enrichment and manipulation, carbon‐based nanoarchitectures provide unique physicochemical advantages for signal amplification, charge transfer, and ultrasensitive biosensing. Their structural characteristics, including surface area, aspect ratio, layer thickness, porosity, defect density, and degree of functionalization, critically determine their electrical conductivity, catalytic activity, biomolecular affinity, and analytical performance. Therefore, rational engineering of carbon nanostructures enables the development of advanced platforms for EVs isolation, molecular profiling, and highly sensitive detection, contributing significantly to biomarker discovery, diagnostics, and therapeutic monitoring [267, 268, 269, 270].

**FIGURE 13:**
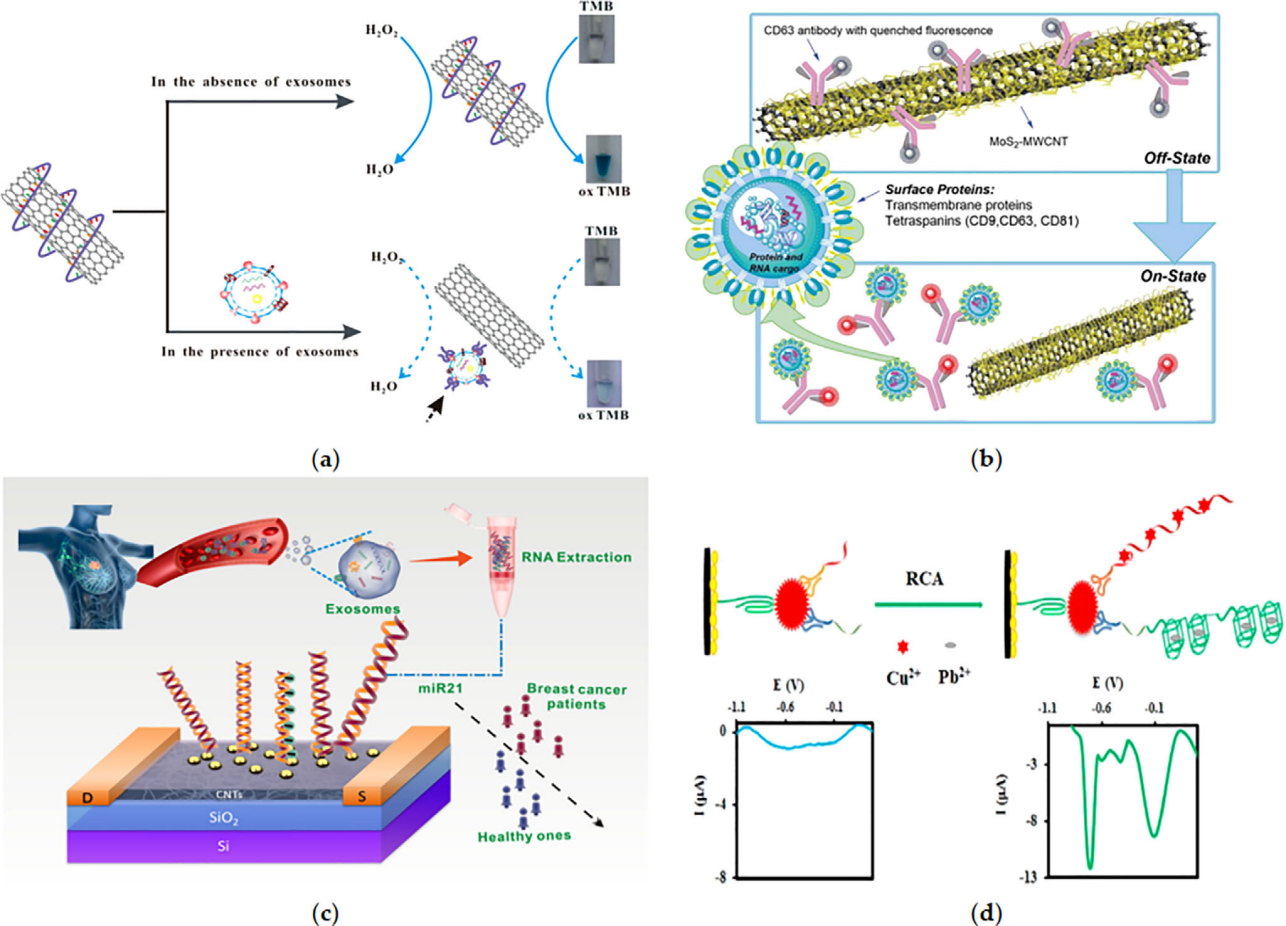
Exosome detecting technique incorporating CNTs. (a) Schematic of a colorimetric aptasensor developed using DNA‐capped SWCNTs for exosome detection. Reproduced with permission from Ref. [271]. Copyright 2017, Elsevier. (b) Schematic of the detection mechanism of a MoS_2_‐MWCNT‐based nanosensor for identification and quantitative analysis of exosomes. Reproduced with permission from Ref. [180]. Copyright 2019, Royal Society of Chemistry. (c) Schematic of the detection procedure of exosomal miRNA‐21 using DNA‐modified CNT FET biosensor. Reproduced with permission from Ref. [272]. Copyright 2021, American Chemical Society. (d) Schematic of an electrochemical aptasensor for the multiplexed detection of exosome biomarkers. Reproduced with permission from Ref. [275]. Copyright 2022, Elsevier.

CNTs, characterized by their tubular SP^2^ structures represent a class of nanomaterials with immense potential in EVs research. Single‐walled carbon nanotubes (SWCNTs) and multi‐walled carbon nanotubes (MWCNTs) are the two leading categories of CNTs, each offering unique properties and applications. One prominent application of CNTs in EVs research is their use in the development of colorimetric sensors for EVs detection. One colorimetric sensor utilizing CD63‐specific aptamer‐conjugated SWCNTs, which exhibit peroxidase activity, resulted in a bright blue color from the TMB solution (Figure 13a) [271]. The aptamers bind with CD63 on exosomes, which caused the exosomes to separate from the SWCNTs when they were exposed to them. This led to a decrease in the peroxidase activity of the SWCNTs and a corresponding decrease in the color intensity of TMB. This sensor enables the detection of exosomes with a LoD of 5.2 × 10^8^ exosomes/mL based on changes in color intensity, offering a simple and accessible method for EVs detection. Moreover, CNTs have been utilized as quenchers in fluorometric nanosensors for sensitive and quantitative detection of exosomes. A nanosensor has been developed employing MoS_2_‐MWCNT modified with R‐phycoerythrin (PE) conjugated CD63 antibody (Figure 13b) [180]. Additionally, exosomes produced from MCF‐7 breast cancer cells could be examined using this method. It was discovered that the fluorophore's recovered fluorescence, which showed a linear response range of 0%–15% v/v (0 to 11.1 × 10^6^ particles/mL) and a LoD of 2.0408 v/v% (14.8 × 10^5^ particles/mL), could be used to evaluate CD63 expression. Furthermore, exosomes with different surface biomarker expressions can be quantified using this nanosensing technique, which has demonstrated that exosomes released from MCF‐7 breast cancer cells express more CD24 than CD63 and CD81. Additionally, CNT‐based field‐effect transistor (FET) biosensors have been constructed for the detection of miRNA from tumor exosomes. Li et al. fabricated an FET biosensor utilizing polymer‐sorted semiconducting CNT films on top of Si/SiO_2_ substrate, followed by deposition of a 3 nm thin film of yttrium via electron beam evaporation (EBE) and oxidizing it to generate an insulating layer (Figure 13c) [272]. Subsequently, gold (Au) was deposited to create AuNPs for anchoring the oligonucleotide probe. Through hybridization, this probe binds to miRNA, causing current changes that allow for the high sensitivity and specificity of exosome identification and quantification, providing a promising tool for disease diagnosis and biomarker detection. Furthermore, CNTs have found applications in electrochemical‐based EVs sensors, such as DNA/ferrocene‐functionalized SWCNTs for the detection of lung cancer exosomes [273]. For the sensitive detection of exosomes, Sahraei et al. devised a biodegradable electrochemical paper‐based device (ePAD) that incorporates synthetic carbon and AuNPs [274]. The surface of the nanocomposites was coated with CD9 antibodies for detection, thus demonstrating remarkable sensitivity and specificity, displaying a LoD of 7 × 10^4^ exosomes/mL, making them valuable tools for EVs analysis in clinical settings. In research conducted by Hashkavayi et al., a screen‐printed carbon electrode was customized with MWCNT, an ionic liquid, and chitosan, and further functionalized with CD63 to facilitate the capture of exosomes (Figure 13d) [275]. Subsequently, EpCAM or HER2 positive exosomes were attached to EpCAM or HER2 aptamers, respectively, incorporating primer sequences that initiated a rolling circle amplification (RCA) reaction. Due to their complexation with the RCA products, the introduction of Cu and Pb ions resulted in robust electrochemical signals that were detected by differential pulse voltammetry (DPV) after RCA.

Graphene, a two‐dimensional sheet of sp^2^ hybridized carbon atoms, has also emerged as a promising platform for EVs recognition and analysis [276, 277]. Graphene‐based FET biosensors functionalized with CD63 antibodies have been exploited for efficient analysis of exosomes in serum samples [278]. The microfluidic channel in the framework divides the graphene into sample‐containing and sample‐free zones, exhibiting distinct electrical properties to sense exosome binding through changes in current and achieved a LoD of 0.1 µg/mL. Another research group devised a reduced graphene oxide (RGO) FET modified with CD63 to identify and detect exosomes, boasting a low LoD of 3.3 × 10^4^ exosomes/mL [279]. This label‐free technique enables direct exosome quantification in serum samples, showing promise for diagnosis of prostate cancer. Moreover, the porous assembly has facilitated exosome isolation both including and excluding additional isolation materials and devices [142, 280, 281]. Similarly, graphitic carbon nitride nanosheets (g‐C_3_N_4_ NSs) have been utilized for colorimetric detection of exosomes based on their peroxidase‐like activities [282, 283, 284]. Wang et al. demonstrated the use of g‐C_3_N_4_ NSs for detecting exosomes by monitoring changes in color intensity, offering a sensitive and selective method for EVs analysis [285]. The research group discovered that ssDNA could get adsorbed on g‐C_3_N_4_ NSs which significantly accelerated their catalytic activity. The maximum reaction rate of H_2_O_2_‐mediated 3,3′,5,5′‐ tetramethylbenzidine (TMB used as a chromogenic substrate) oxidation catalyzed by the ssDNA‐NSs hybrid was at least four times faster than that achieved with unmodified NSs. The high catalytic activity of the ssDNA‐NSs hybrid enabled sensitive colorimetric detection of exosomes when an aptamer against CD63 was incorporated into the hybrid. The sensor was successful in differentiating between the exosomes generated by the MCF‐7 breast cancer cell line and the MCF‐10A control cell line in terms of CD63 expression. As research in this area continues to evolve, carbon nanostructures are expected to play an increasingly important role in unlocking the full potential of EVs for biomedical applications. Despite their advantages, challenges related to reproducibility, biocompatibility, nonspecific interactions, and scalable fabrication remain. Beyond carbon nanomaterials, porous organic frameworks offer further structural tunability through programmable pore architecture and modular chemical functionalities, providing new opportunities for selective EV recognition, capture, and advanced biosensing.

### 4.5 Organic Frameworks

Beyond carbon‐based nanostructures, organic framework materials represent a further evolution of nanoarchitectured platforms by providing precisely tunable pore structures, modular chemical functionalities, and programmable host–guest interactions. Metal–organic frameworks (MOFs) represent a class of versatile 2D and 3D metal–organic crystalline porous nanostructures composed of organic linkers and metal ions, forming highly tunable, porous, and stable structures [285, 286]. Their unique structural characteristics, including high surface area, tunable pore size, abundant active sites, and adjustable metal–ligand coordination environments, enable precise control over molecular recognition, catalytic activity, and signal transduction, making them highly attractive for EVs capture and biosensing applications. These properties have allowed MOFs to be explored extensively in diverse fields, including solar energy conversion, drug delivery, catalysis, and cancer diagnostics [287].

In EVs research, MOFs offer several advantages over conventional nanomaterials because their porous structures can simultaneously facilitate biomarker enrichment, signal amplification, and molecular recognition. The ability to incorporate functional molecules, electroactive species, and affinity ligands within their frameworks provides opportunities to design multifunctional biosensors with improved analytical performance. Consequently, MOF‐based platforms have emerged as promising candidates for electrochemical and optical EVs detection, particularly for point‐of‐care diagnostics and personalized medicine [288]. However, challenges including framework stability in aqueous biological environments, controlled biomolecule accessibility within nanopores, and large‐scale reproducible synthesis remain important considerations for clinical translation.

**FIGURE 14:**
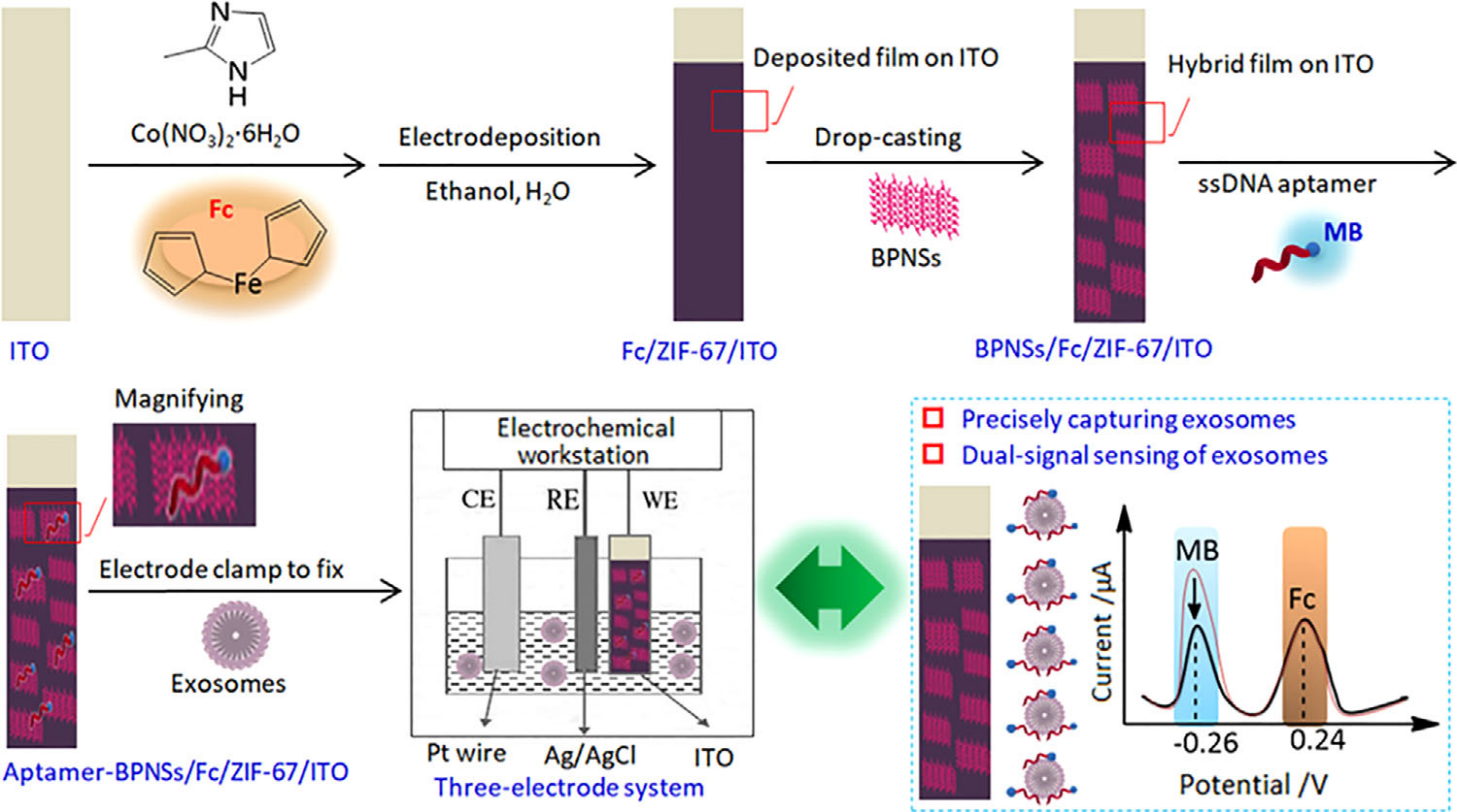
Illustration of the fabrication procedure and application of a dual‐signal and intrinsic self‐calibration aptasensor for exosomes, based on a functional hybrid thin‐film sensing platform aptamer‐BPNSs/Fc/ZIF‐67/ITO. Reproduced with permission from Ref. [197]. Copyright 2020, American Chemical Society.

Utilizing a functional hybrid thin‐film technology, Sun and the team fabricated a unique dual‐signal and intrinsic self‐calibration aptasensor for exosome detection [197]. Black phosphorus nanosheets (BPNSs) and ferrocene (Fc)‐doped metal–organic frameworks (ZIF‐67) were assembled on an indium tin oxide (ITO) slice, after which a ssDNA aptamer tagged with methylene blue was incorporated. The produced aptamer‐BPNSs/Fc/ZIF‐67/ITO platform showed dual redox‐signal responses from Fc (doped into ZIF‐67) and MB (labeled on the aptamer) (Figure 14). The redox current of MB dropped in the presence of particular exosomes produced from cancer cells, whereas that of Fc (as a reference) did not fluctuate. With a LoD of only 100 particles/mL, this novel intrinsic self‐calibration aptasensor was able to identify exosomes with high precision. Moreover, it successfully detected particular exosomes generated from cancer cells in human serum and plasma samples taken from patients with breast cancer as well as healthy controls. Owing to its exceptional capabilities, this aptasensor can be tailored to identify several biomarkers from diverse exosomes generated from cancer cells, thereby improving early cancer screening and clinical diagnosis methodologies.

A highly porous Zr‐based MOFs (UiO‐66‐NH_2_) loaded with methylene blue, a commonly used electroactive redox indicator, were utilized as an electrochemical biosensor for capturing glioblastoma multiforme (GBM) exosomes [194]. A specifically fabricated protein was then manufactured on the electrode surface to identify EGFR and its variant EGFRvIII, which are biomarkers for GBM. This facilitated the capture of GBM exosomes. Subsequently, the MB@Zr‐MOFs attached to the captured exosomes, generating an enhanced electrochemical signal through the interaction between the Zr^4+^ from the MOFs and the phosphate head of the exosomes' lipid membrane. The sensor reached a LoD of 7.83 × 10^6^ exosomes/mL whereas the exosome concentration may be quantitatively analyzed by directly monitoring the electroactive molecules within the MOFs, with a detection range from 9.5 × 10^3^ to 1.9 × 10^7^ particles/µL. Utilizing this sensor, the research group successfully differentiated GBM patients from healthy individuals by detecting and quantifying EGFR/EGFRvIII positive exosomes in serum samples. This sensor exhibited high sensitivity, enabling the detection and quantification of GBM exosomes from serum samples with a low LoD. Furthermore, MOFs have been integrated into portable paper‐based electrochemical biosensors for exosome detection, offering rapid and sensitive analysis. Liu et al. developed a paper‐based sensor by coating Zr‐MOFs on paper surfaces to capture exosomes, followed by the addition of aptamers for specific recognition of exosomal markers [289]. This sensor utilized hybridization chain reaction (HCR) amplification to enhance sensitivity, achieving a low LoD for exosome detection. Additionally, Cao et al. introduced a pH‐sensitive MOF system for electrochemical detection of exosomes, utilizing hyperbranched rolling circle amplification (HRCA) to amplify electrochemical signals [290]. These innovative biosensors offer high sensitivity and specificity, enabling the detection of exosomes with superior performance. Moreover, MOFs offer versatility in signal readout strategies and functionalization approaches for EVs detection, facilitating the development of tailored biosensing platforms for protein and RNA markers [291, 292]. By combining MOFs with other detection techniques, such as magnetic nanoparticles, researchers have achieved efficient capture and purification of exosomes from complex biological samples [293]. For instance, MOF‐based immunoaffinity flakes have been utilized for capturing and separating exosomes in samples obtained from both healthy individuals and patients with lung adenocarcinoma, demonstrating high capture and recovery rates [294].

In parallel with MOFs, covalent organic frameworks (COFs) have recently emerged as another important class of organic framework materials for EVs research. Unlike MOFs, COFs are constructed entirely from covalently bonded organic building blocks, generating highly ordered 2D and 3D crystalline porous structures with excellent chemical tunability and stability [295]. Their metal‐free composition, predictable pore architecture, and rich organic functionality provide additional opportunities for molecular recognition and catalytic signal amplification. Wang et al. showcased the application of COFs in colorectal cancer exosome detection [296]. In this study, they constructed HRPpSC4‐AuNPs@COFs, a novel COF‐based nanoprobe by functionalizing the COF structure with horseradish peroxidase (HRP) and AuNPs treated with *p*‐sulfocalix[4]arene hydrate (pSC4). This nanoprobe could be used to electrochemically identify colorectal cancer‐derived exosomes. With its exceptional conductivity, the AuNPs accelerated charge carrier migration and improved biosensor responsiveness, while the pSC4 served as a flexible linker in this arrangement, recognizing and binding to a variety of amino acid residues on the exosome surface. Furthermore, COFs' high porosity enabled them to load a significant amount of HRP, resulting in strong catalytic activity. Additionally, the COF exoskeleton sustained HRP functionality with noticeably increased stability. With a LoD as low as 160 particles/µL, this novel approach demonstrates outstanding analytical performance for detecting CRC‐derived exosomes over a linear range of 5 × 10^2^ to 10^7^ particles/µL. Furthermore, the approach has demonstrated great potential for clinical diagnosis as it has been successfully used for the analysis of clinical serum samples, efficiently differentiating CRC patients from healthy controls. Despite the advantages of organic framework nanomaterials, including MOFs and COFs, challenges related to biological stability, scalable fabrication, and standardized characterization remain important considerations for clinical translation. Through precise structural design and multifunctional sensing capabilities, organic frameworks represent a significant advancement in nanoarchitectonics for next‐generation EV diagnostics and precision nanomedicine.

## 5 Conclusions and Outlook

Nanoarchitectonics plays a crucial role in the production of EVs, especially exosomes, by providing a platform for precise control over the properties and characteristics of these vesicles. In this context, nanoarchitectonics encompasses various strategies to engineer the environment in which EVs are produced, harvested, and manipulated. Nanoarchitectonics enables the design of specialized bioreactors or culture systems optimized for EVs production. These systems may incorporate nanoscale features such as tailored surfaces, microfluidic channels, or porous scaffolds to enhance cell growth, EVs secretion, and isolation efficiency. For instance, researchers have developed microfluidic devices with nanostructured surfaces to enhance cell growth and EVs secretion, resulting in higher yields of EVs. In addition, nanostructured surfaces can be designed to mimic the extracellular matrix or cellular microenvironment, promoting cell adhesion, proliferation, and EVs release. Surface modifications with specific ligands or functional groups can also facilitate the selective capture or enrichment of EVs from complex biological samples. It has been stated that nanotextured substrates coated with antibodies have been shown to efficiently isolate EVs from biological samples. Moreover, nanoarchitectonics contributes to the fabrication of advanced isolation and purification systems for EVs. This includes the design of nanomaterial‐based separation platforms such as nanoparticles, nanofibers, or nanostructured membranes with high affinity and selectivity for EVs, enabling efficient isolation from biofluids or cell culture media. Nanomaterial‐based separation platforms, such as magnetic nanoparticles functionalized with aptamers, have been employed for rapid and selective isolation of EVs from complex biological fluids.

Nanoarchitectonics enables the loading of therapeutic cargo, such as drugs, nucleic acids, or imaging agents, into EVs for targeted delivery. Nanoparticle‐based carriers or nanoscale delivery platforms can be engineered to encapsulate cargo molecules and facilitate their incorporation into EVs during biogenesis or post‐isolation modification. Lipid nanoparticles containing small interfering RNAs (siRNAs) can be fused with EVs membranes, allowing the efficient loading of siRNAs into EVs for targeted delivery to recipient cells. Nanoarchitectonics facilitates the surface functionalization of EVs with targeting ligands, imaging probes, or stimuli‐responsive moieties for enhanced functionality. This may involve the conjugation of nanoparticles, lipids, or polymers to EVs membranes to impart specific properties or enable controlled release of cargo upon environmental cues. For example, EVs can be decorated with gold nanoparticles conjugated with targeting ligands for enhanced cellular uptake and targeted drug delivery. Furthermore, nanoarchitectonics contributes to the development of nanoscale analytical techniques for characterizing EVs, including size, morphology, surface charge, and cargo content. These techniques, such as nanoparticle tracking analysis (NTA), atomic force microscopy (AFM), or single‐particle interferometric reflectance imaging sensing (SP‐IRIS), provide valuable insights into EV properties and behavior. Overall, nanoarchitectonics plays a multifaceted role in advancing EV production and manipulation, offering opportunities to engineer EVs with tailored properties for a range of biomedical applications, comprising drug delivery, diagnostics, and regenerative medicine. By harnessing nanotechnology principles, researchers can exploit the unique features of EVs and unlock their full therapeutic potential in biomedical research and clinical practice.

While nanoarchitectonics holds great potential in advancing EVs research and therapeutics, there are still a number of challenges to be overcome. To enhance targeted delivery, nanomaterials should be engineered, mainly by optimizing their structure and arrangement, to improve the targeting specificity of EVs, allowing for more precise and efficient delivery of therapeutic substances to diseased cells or tissues. Innovative engineering of EVs likewise involves designing EVs with functionalized surfaces that can interact selectively with target cells or tissues and creating hybrid EVs that combine natural vesicles with synthetic nanoparticles to achieve unique properties and functionalities. Additionally, engineering EVs with nanoarchitectures should enhance their stability and longevity in the bloodstream. Customizable cargo loading is another crucial aspect; nanomaterials should have surfaces optimized or loading specific therapeutic agents. Nanoarchitectonics can also enhance the sensitivity and specificity of EVs‐based diagnostic assays, enabling earlier and more accurate detection of diseases. This enhancement facilitates the easy identification and validation of novel biomarkers within EVs through advanced nanoscale analysis techniques.

Tailoring EVs‐based therapies to individual patients based on their unique biological profiles leverages the precision of nanoarchitectonics, moving toward personalized medicine. Furthermore, establishing standard protocols for the characterization and use of nanoengineered EVs is essential to ensure their efficacy and safety. Additionally, comprehensive studies on long‐term safety, pharmacokinetics, and biodistribution of nanomaterial‐based EVs therapeutics are essential for clinical translation. Collaboration between researchers in nanotechnology, materials science, and biomedical engineering will drive innovation and accelerate the construction of next‐generation EVs‐based therapeutics for a wide range of diseases, including neurological disorders, cancer, and inflammatory conditions. Beyond medicine, exploring the use of nanoengineered EVs in fields such as environmental science, agriculture, and biotechnology holds promise. They can be applied for pollutant monitoring and remediation via targeted delivery of degradative chemicals and enzymes [297]. Furthermore, these engineered EVs have the potential to enhance crop protection and growth by delivering pesticides or growth regulators more efficiently [298]. In conclusion, nanoarchitectonics offers unprecedented opportunities to revolutionize EVs research and therapeutics by providing versatile tools for precise manipulation of EV properties and behavior. By integrating nanomaterials with EVs biology, researchers can overcome key barriers and unlock the full therapeutic potential of EVs for precision medicine applications. Continued interdisciplinary research and technological advancements in nanoarchitectonics will pave the way for transformative breakthroughs in EVs‐based therapeutics and diagnostics.

## Funding

This study was supported by the Cancer Council Queensland through the Next Generation Cancer Council fellowship awarded to M.K.M (CCQ; 2027070). Y.Y. acknowledges financial support from JST‐ERATO Yamauchi Materials Space‐Tectonics Project (JPMJER2003). C.S. is supported by the National Health and Medical Research Council (NHMRC; 1195451). This work used the Queensland node of the NCRIS‐enabled Australian National Fabrication Facility (ANFF).

## Conflicts of Interest

The authors declare no conflicts of interest.

## Data Availability

Data sharing not applicable to this article as no datasets were generated or analyzed during the current study.
